# Multivalent cytomegalovirus glycoprotein B nucleoside modified mRNA vaccines did not demonstrate a greater antibody breadth

**DOI:** 10.1038/s41541-024-00821-3

**Published:** 2024-02-20

**Authors:** Hsuan-Yuan Wang, Leike Li, Cody S. Nelson, Richard Barfield, Sarah Valencia, Cliburn Chan, Hiromi Muramatsu, Paulo J. C. Lin, Norbert Pardi, Zhiqiang An, Drew Weissman, Sallie R. Permar

**Affiliations:** 1 Department of Pediatrics, Weill Cornell Medicine, New York, NY 10065 USA; 2 Duke University Medical Center, Duke Human Vaccine Institute, Durham, NC 27710 USA; 3 Texas Therapeutics Institute, Brown Foundation Institute of Molecular Medicine, The University of Texas Health Science Center at Houston, Houston, TX 77030 USA; 4 Division of Allergy and Clinical Immunology, Department of Medicine, Brigham and Women’s Hospital, Harvard Medical School, Boston, MA 02115 USA; 5 Department of Biostatistics and Bioinformatics, Duke University School of Medicine, Durham, NC 27710 USA; 6 Center for Human Systems Immunology, Duke University Medical Center, Durham, NC 27710 USA; 7 Department of Medicine, Perelman School of Medicine, University of Pennsylvania, Philadelphia, PA 19104 USA; 8 Acuitas Therapeutics, Vancouver, BC V6T 1Z3 Canada; 9 Present Address: Takeda Pharmaceutical, San Diego, CA 92121 USA; 10 Present Address: Department of Microbiology, Perelman School of Medicine, University of Pennsylvania, Philadelphia, PA 19104 USA

**Keywords:** RNA vaccines, Herpes virus

## Abstract

Human cytomegalovirus (HCMV) remains the most common congenital infection and infectious complication in immunocompromised patients. The most successful HCMV vaccine to date, an HCMV glycoprotein B (gB) subunit vaccine adjuvanted with MF59, achieved 50% efficacy against primary HCMV infection. A previous study demonstrated that gB/MF59 vaccinees were less frequently infected with HCMV gB genotype strains most similar to the vaccine strain than strains encoding genetically distinct gB genotypes, suggesting strain-specific immunity accounted for the limited efficacy. To determine whether vaccination with multiple HCMV gB genotypes could increase the breadth of anti-HCMV gB humoral and cellular responses, we immunized 18 female rabbits with monovalent (gB-1), bivalent (gB-1+gB-3), or pentavalent (gB-1+gB-2+gB-3+gB-4+gB-5) gB lipid nanoparticle-encapsulated nucleoside-modified RNA (mRNA–LNP) vaccines. The multivalent vaccine groups did not demonstrate a higher magnitude or breadth of the IgG response to the gB ectodomain or cell-associated gB compared to that of the monovalent vaccine. Also, the multivalent vaccines did not show an increase in the breadth of neutralization activity and antibody-dependent cellular phagocytosis against HCMV strains encoding distinct gB genotypes. Interestingly, peripheral blood mononuclear cell-derived gB-2-specific T-cell responses elicited by multivalent vaccines were of a higher magnitude compared to that of monovalent vaccinated animals against a vaccine-mismatched gB genotype at peak immunogenicity. Yet, no statistical differences were observed in T cell response against gB-3 and gB-5 variable regions among the three vaccine groups. Our data suggests that the inclusion of multivalent gB antigens is not an effective strategy to increase the breadth of anti-HCMV gB antibody and T cell responses. Understanding how to increase the HCMV vaccine protection breadth will be essential to improve the vaccine efficacy.

## Introduction

Human cytomegalovirus (HCMV), a ubiquitous β-herpesvirus, is a common cause of mild to severe disease in immunocompromised hosts and infants born with congenital infection^1,2^. The frequent and severe impacts of this infection have led to an HCMV vaccine being listed as a Tier 1 priority vaccine by the National Academy of Medicine for over 20 years^3^. HCMV glycoprotein B (gB), a relatively conserved membrane glycoprotein essential for viral entry into all host cells, has been identified as a vaccine target since the 1980s due to its ability to elicit both neutralizing and non-neutralizing antibody responses^4–8^. Previously, a gB subunit vaccine combined with the squalene adjuvant MF59, demonstrated modest effectiveness (~50%) against virus acquisition in phase II clinical trials^9–12^. Yet, the efficacy achieved with the HCMV gB-based vaccine will need to be enhanced in future vaccine products for clinical translation.

Vaccine immunogenicity and breadth have been increased by the inclusion of multiple antigen valences and serotype-specific immunogens for bacteria and viruses^13^. Some examples of clinically available multivalent vaccines include the poliovirus vaccine, pneumococcal vaccine, and human papillomavirus vaccine^12^. HCMV gB, has been determined to have 5 main gB genotypes: gB-1, gB-2, gB-3, gB-4, and gB-5. All gB genotypes were reported to share 96% mean conserved identity at amino acid level^14,15^. The most variable regions within the 5 genotypes are codons 26–70, which include antigenic domain 2 (AD-2) site 2 and partial AD-2 site 1, and codons 441–490 that cover the furin cleavage site^16–18^ (Supl. Fig. 1A, C). A recent study analyzing the gB genotype variation between placebo recipients and vaccinees who received the gB/MF59 vaccine, which is based on the Towne strain (gB-1), showed that vaccinees were more likely to be infected with an HCMV strain encoding gB-3 and gB-5 compared to HCMV strains encoding gB-1, gB-2, and gB-4 genotypes via full gB open reading frame (ORF) next-generation sequencing (Supl. Fig. 1B)^19^. Yet, a previous report that examined the gB genotype of HCMV infections in this vaccine trial via Sanger sequencing did not reflect these findings^20–22^.

Lipid nanoparticles-encapsulated nucleoside-modified mRNA (mRNA–LNP) is a novel vaccine platform that was translated for safe and effective human use in the severe acute respiratory syndrome coronavirus 2 (SARS-CoV-2) pandemic^23^ and has had success in other vaccine pursuits, including human immunodeficiency virus type 1 (HIV-1)^24,25^, Zika virus^26^, influenza virus^27^, and herpes simplex virus type 2 (HSV-2)^28^. Additionally^29^, multivalent mRNA–LNP vaccines have proved to be a promising strategy for the development of broadly protective SARS-COV-2 and influenza virus vaccines^30–33^. Recently, ModernaTX, Inc. has launched a phase III trial of an mRNA–LNP including HCMV Merlin strain gB and the pentameric glycoprotein complex. Pre-clinical testing of the multivalent vaccine in mice and non-human primate models (NCT05085366) demonstrated robust immunogenicity and epithelial cell neutralization responses^34,35^. Our lab previously showed that the mRNA–LNP vaccine encoding full-length gB-1 enhanced the durability and breadth of peptide-binding responses compared to the most effective vaccine to date, the gB/MF59 vaccine^36^.

Here, we compare the immunogenicity of monovalent (gB1), bivalent (gB1 + gB3), and pentavalent (gB1 + gB2 + gB3 + gB4 + gB5) HCMV gB mRNA–LNP vaccines using the New Zealand White rabbit model. Our goal was to evaluate whether a vaccine including multiple gB genotypes could elicit broader humoral immunity against multi-genotypic HCMV infections compared to the monovalent gB immunogen. The findings in this study will be important to guide whether including multiple gB genotypes in the HCMV vaccine will result in an increased breadth of immune response towards gB.

## Results

### Study design and immunization schedule

Before the in vivo study, we compared the in vitro transfection of mRNA–LNPs encoding each gB genotype individually to determine the antigenicity of each gB antigen. The mRNA–LNPs encoding each gB genotype were co-transfected with green fluorescence (GFP) DNA plasmid in HEK 293 T cells and incubated with polyclonal IgG, HCMV immune globulin (CytoGam) and seronegative plasma. The polyclonal IgG binding to cell-associated gBs was measured by flow cytometry (Supl. Fig. 2A–C). Both CytoGam and HCMV-seronegative plasma showed similar binding to the five cell-associated gB antigens, suggesting similar antigenicity among the five gB antigens.

**Fig. 1:**
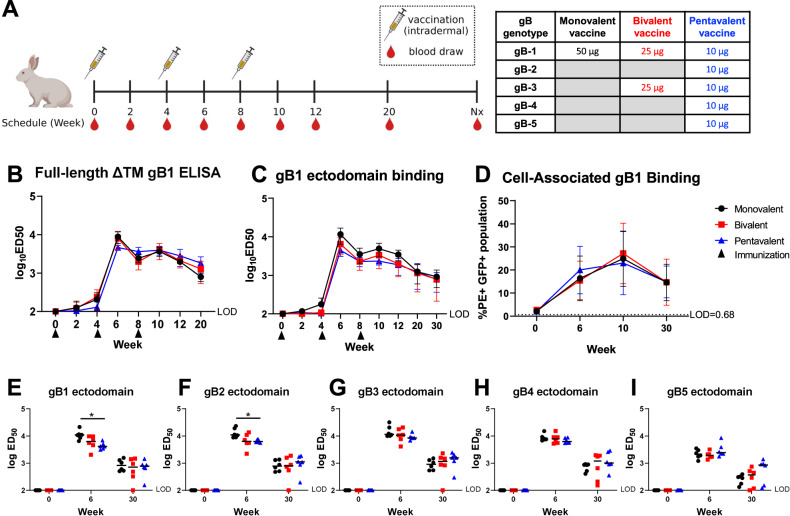
Monovalent, bivalent, and pentavalent gB mRNA–LNP vaccinated rabbit plasma IgG binding to soluble and cell-associated gB, and gB-specific IgG binding breadth. **A** Rabbit gB mRNA–LNP vaccination schedule. 18 rabbits were divided into three groups to receive gB mRNA monovalent, bivalent, and pentavalent vaccines, respectively, at week 0, 4, and 8. All three vaccines include a total of 50 µg 1-methylpseudouridine-modified gB mRNA while encoding single or multiple gB genotypes in each vaccine (Monovalent: 50 µg gB-1 mRNA; Bivalent: 25 µg gB-1 + 25 µg gB-3 mRNA; Pentavalent: 10 µg gB-1 + 10 µg gB-2 + 10 µg gB-3 + 10 µg gB-4 + 10 µg gB-5 mRNA.) Blood samples were collected every 2 weeks between week 0 and week 12, at week 20, and during necropsy. 15 animals underwent necropsy at week 30. The other 3 animals (1 animal from each group) received an extra boost at week 41 to prepare rabbit PBMCs for the functional antibody screening and underwent necropsy (Nx) at week 43. This figure was created with Biorender.com. **B**–**D** The dynamics of rabbit plasma IgG binding magnitude to soluble full-length gB-1 with the deletion of transmembrane domain (**B**) and soluble gB-1 ectodomain (**C**) was measured by ELISA, and the IgG binding to cell-associated full-length gB-1 (**D**) for the pre-immune (week 0), peak immunogenicity (week 6 and 10), and durability (week 30) timepoints was estimated by gB-transfected cell binding assay. Data points are shown as the average IgG binding response with one standard deviation. A statistically significant difference was observed in the binding to the soluble gB-1 ectodomain (*p*-value 0.002, FDR-adjusted *p*-value of 0.006). **E**–**I** Rabbit plasma IgG binding breadth to soluble gB ectodomains encoding 5 genotypes: gB-1 (**E**), gB-2 (**F**), gB-3 (**G**), gB-4 (**H**), and gB-5 (**I**) for the pre-immune (week 0), peak immunogenicity (week 6), and durability (week 30) timepoints was assessed by ELISA. Each data point represents the IgG binding response of one individual animal, with the median response labeled by a black line. Black circles: rabbits immunized with monovalent vaccine (gB-1); red squares: those immunized with bivalent vaccine (gB-1 + gB-3); blue triangles: those immunized with pentavalent vaccine (gB-1 + gB-2 + gB-3 + gB-4 + gB-5). We first performed a multivariate Kruskal–Wallis (with *p*-values calculated via permutation). We observed a significant association at week 6 and then followed up with Kruskal–Wallis tests at each gB. * indicate where there was a significant post-hoc association at week 6 (multiple testing via Holm adjusted *p*-value < 0.10.). Week 0 was not analyzed.

The immunization schedule is shown in (Fig. 1A). A total of 18 rabbits were divided into three groups (*n* = 6 in each group) to receive gB mRNA–LNP monovalent, bivalent, and pentavalent vaccines, respectively, at weeks 0, 4, and 8. These gB mRNA–LNP vaccines were given intradermally since this route showed the longest duration of mRNA translation and half-life compared to other routes^29^. To prevent the structural interference of the multivalent mRNA–LNP vaccines at the injection sites and to prevent administration differences among the three groups, each rabbit received their vaccine doses split across six injection sites.

All three vaccines include a total of 50 µg (~0.025 mg/kg) 1-methylpseudouridine-modified gB mRNA encoding single or multiple genotypes in each vaccine (Monovalent: 50 µg gB-1 mRNA; Bivalent: 25 µg gB-1 + 25 µg gB-3 mRNA; Pentavalent: 10 µg gB-1 + 10 µg gB-2 + 10 µg gB-3 + 10 µg gB-4 + 10 µg gB-5 mRNA). The monovalent vaccine encodes gB-1 antigen, the gB genotype that was included in the most efficacious HCMV gB vaccines to date, gB/MF59 and mRNA-1647 vaccines^9,34,35^. Among the five gB genotypes, gB-1, gB-2, and gB-3 are more prevalent compared to gB-4, and gB-5^15^. Since gB-3 and gB-5 genotypes were reported to share a greater genetic similarity compared to gB-1, gB-2, and gB-4^4,20^, we chose to include the most prevalent two genotypes that are located at the tips of two subtrees of the phylogenetic tree, gB-1 and gB-3 (Supl. Fig. 1B).

We determined the total vaccine dosage by giving New Zealand White rabbits 50 µg mRNA–LNP vaccines based on previous studies^24,27^. Since multivalent vaccines were usually designed by equally distributing the total dosage for each antigen at a 1:1 ratio^30–33^, we applied a similar strategy to design the bivalent and pentavalent gB mRNA vaccines in our study.

### Rabbit plasma gB-specific IgG binding to soluble gB protein, cell-associated gB, and linear gB peptide

We first measured the dynamics of rabbit plasma IgG binding to soluble full-length gB-1 without the transmembrane domain (ΔTM gB-1) and gB-1 ectodomain, matched to the monovalent vaccine strain (Fig. 1B, C). As expected, the IgG binding to both full-length gB-1 and gB-1 ectodomain peaks at weeks 6 and 10, 2 weeks post the 2^nd^ and 3^rd^ boost, respectively. The gB-specific IgG binding response waned gradually after weeks 6 and 10. Interestingly, although the bivalent and pentavalent vaccines include less gB-1 mRNA–LNP compared to the monovalent vaccine, no significant differences (*p*-value < 0.05 and multiple-testing corrected *p*-value < 0.1) of the IgG binding magnitude to the soluble and cell-associated full-length gB-1 among three vaccine groups were observed (Fig. 1B). Using a longitudinal non-parametric comparison, we saw a statistically significant gB-1 ectodomain IgG binding across time between the three vaccine groups (*p*-value 0.002, pFDR 0.006). It appears that at peak immunogenicity (weeks 6 and 10), the monovalent vaccine elicited the highest binding response (Fig. 1C).

We next evaluated whether the bivalent and pentavalent vaccines increase the IgG binding breadth by comparing the rabbit plasma IgG binding to soluble gB ectodomains of the five genotypes at week 0 (pre-vaccination), 6 (peak immunogenicity), and 30 (durability timepoint) (Fig. 1E–I). We observed a statistically significant association at gB-1 and gB-2 (unadjusted *p*-value < 0.05, multiple testing Holm-adjusted *p*-value < 0.1), driven by the higher IgG response elicited by the monovalent vaccine. At week 30 (durability timepoint), although the pentavalent vaccine elicited a slightly higher IgG binding to gB-5 ectodomain in some animals compared to the monovalent and bivalent vaccines, no significant differences among the three groups emerged.

A previous study from our lab demonstrated that the gB/MF59 vaccine protection against HCMV acquisition was associated with the level of plasma IgG binding to cell-associated gB. It was also shown that the conformation of gB expressed in soluble form is different from that expressed on cell surface^5^. Thus, we also assessed the dynamics of rabbit plasma IgG binding to cell-associated gB-1 (Fig. 1D) and the other gB genotypes (Supl. Fig. 3C–E). To measure the IgG binding to cell-associated gB of different genotypes, we designed GFP-tagged plasmids that include full-length gB sequences encoding the five genotypes and T2A peptide to prevent the structural interference between gB and GFP (gB-T2A-GFP plasmid). The IgG binding to cell-associated gB-1 peaked at week 10, 2 weeks post the 3^rd^ boost. No significant differences in the plasma IgG binding kinetics to cell-associated gB-1 were observed among three groups (Fig.1D). Due to the varied transfection efficiency, the IgG binding to the five gB genotypes at weeks 6, 10, and 30 was normalized to that at week 0 (Supl. Fig. 3C–E). Overall, the bivalent and pentavalent vaccines did not increase the IgG binding breadth to cell-associated gB encoding the five genotypes compared to the monovalent vaccine as we expected.

Further, we measured the rabbit plasma IgG binding response to linear, overlapping 15-mer gB peptides covering the variable region, amino acid codons 1–77 of gB-1 or gB-2/3 genotypes (27 unique peptides) (Supl. Table 1). We designed peptides based on the monovalent vaccine-matched genotype, gB-1, and the genotypes only included in the bivalent and pentavalent vaccine, gB-2/3, since gB-2 and gB-3 sequences share the greatest similarity compared to other genotypes in this region (Supl. Fig. 1A). The gB peptide-binding IgG response of the monovalent, bivalent, and pentavalent vaccinated rabbit plasma at week 10 revealed no observable differences comparing the number of peptides bound by plasma IgG from gB-1 or gB-2/3 among three vaccine groups (Supl. Fig. 4A–C). The bivalent and pentavalent gB mRNA LNP vaccines had similar IgG binding breadth compared to the monovalent mRNA LNP vaccine.

### Neutralization and antibody-dependent cellular phagocytosis (ADCP) responses

With the incorporation of multiple gB genotypes in the multivalent vaccines, we hypothesized that bivalent and pentavalent vaccines could elicit a broader functional antibody response, such as neutralizing antibody and antibody-dependent phagocytosis (ADCP) response against HCMV strains encoding different gB genotypes.

**Fig. 2:**
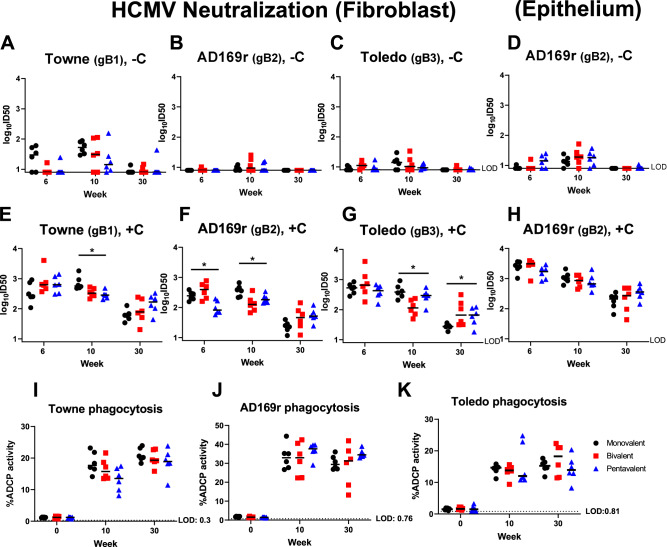
Monovalent, bivalent, and pentavalent gB mRNA–LNP vaccine elicit similar magnitude and breadth of HCMV-neutralizing antibody response against HCMV strains with gB genotype 1, 2, or 3. Rabbit plasma IgG neutralization of HCMV Towne strain encoding gB-1 (**A**, **E**), AD169r strain encoding gB-2 (**B**, **D**, **F**, **H**), and Toledo strain encoding gB-3 (**C**, **G**) without the addition of purified rabbit complement (−C) (**A**–**D**) or with the purified rabbit complement (+C) (**E**–**H**) was estimated in fibroblasts (**A**–**C**, **E**–**G**) or epithelial cells (**D**, **H**)). We did not test the complement data (−C, Fig. 2A–D). *P < 0.1, Kruskal–Wallis test performed with the multiple testing correction via FDR. Post-hoc tests were done via the Wilcoxon Rank Sum Test to assess for differences between pairs of vaccine groups. Phagocytosis of the whole HCMV virion of the Towne strain (**I**), AD169r strain (**J**), and Toledo strain (**K**) were measured by flow cytometry. Antibody neutralization and phagocytosis activity were assessed at the pre-immunization (week 0), peak immunogenicity (week 6, 10), and durability (week 30) timepoints. Each data point represents the neutralization activity or whole virion phagocytosis response of one individual animal, with the median response labeled by a black line. Black circles: rabbits immunized with monovalent vaccine; red squares: those immunized with bivalent vaccine; blue triangles: those immunized with pentavalent vaccine. Kruskal–Wallis test was performed with multiple testing corrections by FDR. Post-hoc comparisons were done for (**E**–**H**) (Wilcoxon Rank Sum Test *p < 0.1).

We first investigated the vaccine-elicited neutralizing antibody response against Towne (gB-1), AD169r (gB-2), and Toledo (gB-3) strains in fibroblasts (Fig. 2A–C, E–G) and epithelial cells (Fig. 2D, H). A previous study showed that complement enhances and can be required for the neutralizing potency of gB-specific antibodies. Therefore, we assessed the vaccine-elicited neutralizing antibody response with and without rabbit complement^37^. As expected, the neutralizing titer without rabbit complement was lower and mostly undetectable against AD169r and Toledo strain compared to that performed with rabbit complement (Fig. 2E–H). Therefore, we did not perform the statistical analysis on the neutralizing antibody titer data generated without rabbit complement.

We utilized a Kruskal–Wallis test (*p*-values determined via 100,000 permutations) to determine whether the neutralizing antibody response with rabbit complement among three vaccine groups is different. At peak immunogenicity (week 6 or 10), the monovalent vaccine group exhibited a higher or similar neutralization capability against Towne, AD169r, and Toledo strains compared to the multivalent vaccine groups by the multiple testing correction analysis. After performing the post-hoc analysis (15 Wilcoxon Rank Sum tests), we observed that the monovalent vaccine elicited a statistically higher neutralizing response at week 10 against Toledo strain (*p*-value 0.0043, pHolm 0.065) than the bivalent vaccine and against Towne strain (*p*-value 0.0043, pHolm 0.065) than the pentavalent vaccine. No statistical differences in the neutralizing antibody responses in epithelial cells against the AD169r strain, which was repaired in the ULb’ region to maintain epithelial cell tropism, were observed. These results suggest that the bivalent and pentavalent vaccines did not elicit a broader neutralizing antibody response at peak immunogenicity.

Interestingly, we observed a statistical difference at a durability time point (week 30) against the Toledo strain (a gB-3 HCMV strain included in the bivalent and pentavalent vaccine design) in the presence of rabbit complement in fibroblasts. Due to the sample size and number of post-hoc tests, the association appeared to be driven by monovalent versus pentavalent (*p*-value 0.065, pHolm 0.39) and monovalent versus bivalent (*p*-value 0.0087, pHolm 0.11), though neither were significant after adjustment for multiple testing. We also performed a secondary analysis by Wilcoxon rank sum test to compare the monovalent vaccine to the multivalent vaccines (combine bivalent and pentavalent vaccine groups) and observed a statistically higher neutralizing titer against AD169r strain in fibroblasts at week 30 (not indicated in the figure).

Subsequently, we examined whether a multivalent gB mRNA vaccine increased the breadth of non-neutralizing antibody functions since multiple previous studies reported that gB variable regions are major non-neutralizing antibody epitopes^36,38^. Our lab previously showed that gB-specific antibodies elicited by the gB/MF59 vaccine-mediated robust ADCP response against whole HCMV virions at a similar level to those elicited by chronically HCMV-infected individuals^4^. In addition, high magnitude ADCP response against HCMV virions was shown to be associated with a lower risk of congenital HCMV transmission in seropositive women^39^. We measured the rabbit plasma ADCP response against the whole HCMV virions from Towne (gB-1), AD169r (gB-2), and Toledo (gB-3) strains (Fig. 2I–K). The anti-HCMV ADCP potency of vaccinated-rabbit plasma IgG peaked at week 10 and showed a durable response until week 30. However, the bivalent and pentavalent vaccines did not elicit a higher ADCP response against HCMV virions encoding gB-1, gB-2, or gB-3 as we expected. Therefore, our data suggest the multivalent vaccines might not show an advantage for the breadth of this key Fc-mediated effector function.

### Monoclonal antibody isolation from rabbit memory B cells

**Fig. 3:**
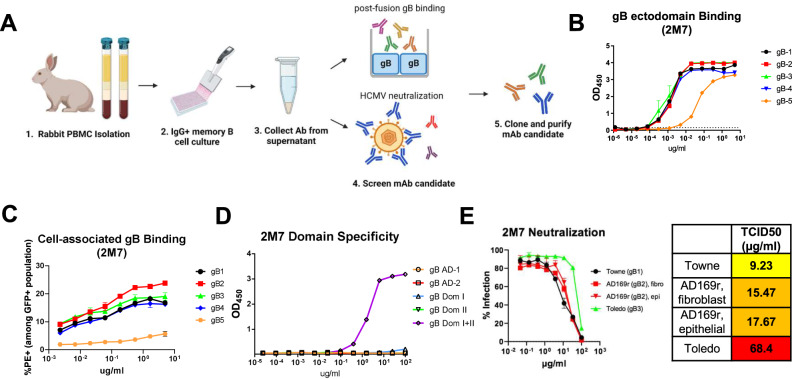
Genotype-dependent binding and neutralization properties of a gB-specific monoclonal antibody isolated from transformed memory B cells of multivalent gB mRNA–LNP-immunized rabbits. **A** gB-specific mAb was isolated from rabbit PBMCs memory B cell culture. Rabbit PBMCs at peak immunogenicity (week 10) were isolated from EDTA whole blood, and the IgG+ memory B cells were cultured. The memory B cell culture supernatant was collected and performed post-fusion gB binding ELISA or fibroblast neutralization against AD169r strain for screening. The mAb candidate with potent gB-binding was cloned and purified for the downstream characterization. **B**, **C** Isolated gB-specific mAb 2M7 binding to soluble gB ectodomains encoding 5 genotypes was measured by ELISA (**B**), and cell-associated full-length gB encoding 5 genotypes was estimated by flow cytometry (**C**). 2M7 binding to gB genotypes is color-coded in lines: black, gB-1; red, gB-2; green, gB-3; blue, gB-4; orange, gB-5. **D** 2M7 binding strength to gB AD-1, AD-2 site 1, Domain I, Domain II, and Domain I + II was measured by ELISA, demonstrating conformational-dependent Dom I + II specificity. 2M7 binding to gB domains is color-coded with symbols: black circle, AD-1; red square, AD-2 site 1; green upward triangle, Domain I; blue downward triangle, Domain II; orange diamond, Domain I + II. **E** The neutralization capability of 2M7 against HCMV Towne strain (gB-1) and Toledo strain (gB-3) was estimated in fibroblasts, and that against AD169r strain (gB-2) was measured in fibroblasts and epithelial cells. 2M7 neutralization is color-coded based on the HCMV strains: black, Towne strain; red, AD169r strain; green, Toledo strain.

After characterizing mono- and multivalent vaccine-elicited plasma IgG binding and functional antibody responses, we investigated whether rabbits immunized with the multivalent vaccines could generate gB genotype-specific monoclonal antibodies (mAbs). We performed single memory B cell culture from rabbit peripheral blood mononuclear cells (PBMCs) at week 10 (2 weeks post 3rd boost) to isolate gB-specific mAbs, using methods reported previously^40^. Due to the limited cell numbers, we mixed two rabbit PBMCs samples from the monovalent and pentavalent groups to perform a single memory B cell culture. From the 600 single memory B cell cultures, we identified 38 mAbs in culture supernatant that bound to full-length gB-1 (Supl. Fig. 6A, B) and 1 of those 38 (mAb 2M7) potently neutralized HCMV in fibroblasts against AD169r strain. The potent neutralizing mAb 2M7 was further cloned and purified for the downstream antibody characterization (Fig. 3A).

We characterized the genotype-dependent binding and neutralization property of mAb 2M7 against HCMV strains encoding different gB genotypes (Fig. 3B–E). The mAb 2M7 demonstrated a similar binding to gB ectodomain or cell-associated gB encoding genotypes 1–4, but a lower binding to gB-5 (Fig. 3B, C). We further characterized the epitope binding specificity of 2M7 and found that 2M7 demonstrated high binding to soluble gB domain I + II construct but not to AD-1, AD-2, domain I, and domain II, individually (Fig. 3D). Further, 2M7 exhibited the highest neutralizing potency against the HCMV Toledo strain (gB-3) when compared to the Towne (gB-1) and AD169r (gB-2) strains (Fig. 3E). These results demonstrate that the mAb 2M7 possess a gB genotype-dependent function.

### Vaccine-elicited gB-specific PBMC and splenic T-cell response

**Fig. 4:**
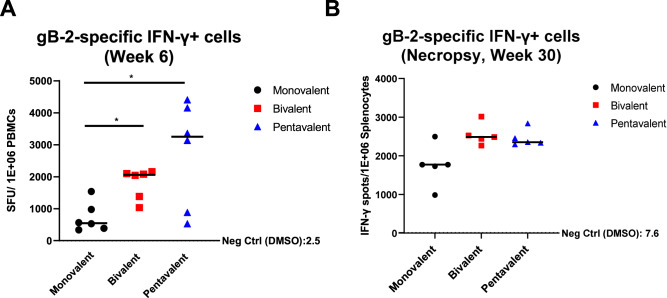
Multivalent gB mRNA–LNP vaccine elicited a stronger gB-2-specific (AD169r strain) IFN-r+ cell response in PBMCs at peak immunogenicity and in spleen at necropsy than monovalent gB mRNA–LNP vaccine. gB-2-specific IFN-r+ cells from rabbit PBMCs or splenocytes were measured by ELISPOT. Full-length, overlapping gB-2 peptide pool stimulation was performed in duplicate. **A** Quantification of IFN-r+ spots from rabbit PBMCs stimulated with gB-2 peptides at peak immunogenicity (Week 6) from monovalent, bivalent, and pentavalent vaccine groups. **B** Quantification of IFN-r+ spots from rabbit splenocytes stimulated with gB-2 peptides at necropsy from monovalent, bivalent, and pentavalent vaccine groups. Fifteen animals underwent necropsy at week 30, while 3 animals (1 animal from each group) underwent necropsy at week 43 due to an extra boost prior to B cell isolation at week 41. Each data point represents one peptide-stimulated well from individual animals, with the lines designating the median. The assay limitation was determined by the average spot count of the cells incubated with DMSO only (negative control). Black circles: rabbits immunized with monovalent vaccine; red squares: those immunized with bivalent vaccine; blue triangles: those immunized with pentavalent vaccine. Kruskal–Wallis test with Bonferroni correction. An association was observed at week 6 (unadjusted *p*-value of 0.025). Post-Hoc tests via Wilcoxon Rank Sum test were then done at week 6 (*p < 0.05, **p < 0.01, ***p < 0.001), revealed it was largely driven by differences between Monovalent and the two other vaccine groups.

T cell immunity is crucial for controlling HCMV infection and generating broad antibody response, which is commonly assessed in HCMV vaccine candidate studies^41,42^. An HCMV vaccine that can elicit a broad T-cell response against multiple HCMV strains would also be desirable. We hypothesized that the pentavalent vaccines could elicit a more robust T cell response against a gB genotype not included in the monovalent or bivalent vaccine, for instance, gB-2. To test our hypothesis, we stimulated rabbit PBMCs at peak immunogenicity (week 6) and splenocytes at necropsy (week 30) with HCMV gB-2 peptide pools and measured the IFN-γ-secreting cell numbers by ELISPOT (Fig. 4A, B, Supl. Fig. 8A, B). Three animals (one animal from each group) underwent necropsy at week 43 rather than week 30 due to an extra boost for B cell isolation at week 41. The week 43 splenocytes were stimulated with HCMV gB-2 peptide pools but not included in the statistical analysis (Supl. Fig. 8C).

The pentavalent-vaccinated rabbit PBMCs demonstrated the highest IFN-γ T cell response against gB-2 at week 6, followed by those from the bivalent and monovalent vaccine groups (Fig. 4A). This result is consistent with our expectation that the pentavalent vaccine is the only vaccine that includes gB-2 immunogen. By performing the post-hoc analysis, a statistically higher IFN-γ-secreting T cell response was observed comparing the bivalent and monovalent, the pentavalent and monovalent vaccine groups, respectively. No statistical significance of IFN-γ-secreting splenic T cell response against gB-2 was observed at week 30. Interestingly, the bivalent vaccine elicited a higher IFN-γ splenic T cell response against gB2 at week 6 than the monovalent vaccine group (Fig. 4B), despite that gB2 was not a component of either vaccine.

To further investigate whether the multivalent vaccines increase the T cell breadth targeting the gB variable regions, we designed two 15-mer peptide pools with 11 overlapping amino acids targeting variable regions of gB-3 and gB-5 genotypes (codons 25–70, and 447–490) and repeated the IFN-γ ELISpot assay with splenocyte samples collected at week 30 (Sup Table 2). The bivalent and pentavalent vaccine elicited a higher gB-3-specific but not gB-5-specific IFN-γ+ cell response, though none of the responses showed statistical differences (Supl. Fig. 9A, B).

## Discussions

Our study is the first to investigate whether the inclusion of multiple gB genotypes in HCMV vaccine design would increase the breadth of humoral and T cell responses against multiple HCMV strains. Applying the recently translated and highly successful nucleoside-modified mRNA–LNP platform, we vaccinated three groups of rabbits with the monovalent (gB-1), bivalent (gB-1, gB-3), and pentavalent (gB-1, gB-2, gB-3, gB-4, and gB-5) gB vaccines, respectively, and compared the vaccine immunogenicity. The kinetics of rabbit plasma IgG binding response against full-length soluble and cell-associated gB-1 post-immunization was similar among the three groups. Yet, the multivalent vaccine groups did not demonstrate a broader IgG response against soluble and cell-associated gB encoding the five genotypes or enhance the breadth of functional antibody responses. Interestingly, the multivalent vaccines elicited a higher T cell magnitude than the monovalent vaccine against the monovalent and bivalent vaccine-mismatched gB genotype (gB-2) at peak immunogenicity, though the multivalent vaccines did not increase the T cell breadth against gB-3 and gB-5 variable regions. These results indicated the inclusion of multiple gB antigens in the HCMV vaccine is not an effective strategy to increase the humoral and T cell response breadth.

We designed an HCMV vaccine to include the full-length gB mRNA rather than only include the variable regions to maximize the immunogenicity since there were six antigenic domains (AD) identified across the HCMV gB sequence^36,38,43–46^. We chose to test the multivalent gB mRNA vaccine efficacy using a rabbit model as we previously validated that the in vitro assays could be applied to measure the vaccine-elicited functional antibody responses and epitope specificity^47^. Also, we chose to immunize rabbits intradermally based on the previous mRNA–LNP vaccine route of administration study that the intradermal route led to the longest mRNA translation and duration^29^. The intradermal route of administration has also been applied in multiple mRNA–LNP vaccine studies in rabbits^24,27^.

Our data showed that the pentavalent vaccine included only a fifth of the total gB-1 mRNA–LNP dose compared to that of the monovalent vaccine, and elicited a similar binding and functional antibody response to the monovalent vaccine. The multivalent vaccines did not increase the breadth of IgG binding or functional antibody responses as we expected, suggesting that the gB mRNA–LNP vaccine-elicited humoral response mainly targets the conserved domains instead of variable regions on the gB sequence. Additionally, the structural differences among the five gB genotypes may be subtle, so the multivalent vaccines did not elicit a polyclonal humoral response that had distinct breadth from the monovalent vaccine. This conclusion could be supported by ~96% mean conserved identity at amino acid level among gB genotypes^14^. Currently, only a gB-2 (AD169r) ectodomain post-fusion structure and a gB-1 (Towne) pre-fusion structure have been published^48^. Although multiple studies regarding gB genotyping have been widely published^15^, the structural differences among gB genotypes, particularly the flexible AD2 domain, remain elusive.

Interestingly, while the multivalent gB vaccines did not elicit a polyclonal response that had distinct breadth from the monovalent vaccine, the recognition of distinct gB genotypes was demonstrated on the mAb level. Previously, we have compared the binding preference of gB-specific antibodies, isolated from HCMV-infected individuals to the five genotypes^22^. In this study, we isolated a gB-specific neutralizing antibody 2M7 from vaccinated rabbit PBMCs that demonstrated genotype-dependent function. The mAb 2M7 showed a similar binding to soluble and cell-associated proteins encoding gB-1, gB-2, gB-3, and gB-4 genotypes, but a lower binding to gB-5 soluble and cell-associated proteins. This finding suggests the gB-5 protein structure might be the most dissimilar among the five genotypes, even though gB-3 and gB-5 share a greater genetic similarity in the amino acid sequence^19,20^. The possible unique mutations in the domain I + II region in gB-5 (compared to gB-1) include K112V, T161S, V200L, P218S, R246H, I257M, T259A, L309Q, E364K, L456A, R450S, T451K, K452R, G457N, S474L, and M476N. Additionally, 2M7 neutralized Toledo strain (gB-3) the most potently, although also neutralized Towne (gB-1) and AD169r (gB-2) strains. The binding and neutralization profile suggests that 2M7 might target variable regions of gB. Therefore, we characterized the 2M7 binding domain specificity and found that 2M7 exhibited the strongest binding to gB Dom I + II but no binding to individual AD-1, AD-2, Dom I, and Dom II. Concordant with a previous study, gB Dom I and Dom II were found to be the major targets of gB-specific neutralizing antibodies^36^. Further characterization of gB-specific genotype-dependent neutralizing antibodies might direct the future HCMV vaccine design.

Our data also showed that the multivalent vaccines elicited a higher gB-2-specific T-cell response in PBMCs compared to that of the monovalent vaccine, which did not contain the gB-2 antigen (Fig. 4A). As expected, the pentavalent vaccine elicited the highest IFN-γ-secreting T cell response among the three vaccine groups at peak immunogenicity. Yet, surprisingly, the bivalent vaccine, which did not contain the gB-2 antigen, also demonstrated a higher T cell response than the monovalent vaccine at peak immunogenicity and durability timepoints. Considering the inclusion of gB genotypes in the monovalent (gB-1) and bivalent (gB-1 and gB-3) vaccines, this observation might indicate the T cell epitopes of gB-2 and gB-3 genotypes are somewhat similar compared to gB-1. The sequence alignment suggests that gB-2 and gB-3 are almost identical at codon 28–70 while gB-1 has completely distinct codons at the same region (Supl. Fig. 1A). Therefore, the codon 28–70 region potentially contains a T cell epitope target, although two previous studies showed the first 80 amino acids in gB are not immunodominant T cell epitopes in HCMV-seropositive healthy donors^49,50^. The multivalent vaccine groups showed a higher gB-3-specific but not gB-5-specific IFN-γ+ cell response, though no statistical differences were reported (Supl. Fig. 9A, B). The variable responses within the animals from the same group could be explained by the diverse genetic differences of the outbred rabbit stocks. Alternatively, this result might suggest the multivalent vaccine-elicited T-cell response targets the conserved domains instead of variable domains on gB. Further studies are required to identify how to increase T cell breadth in HCMV vaccine design.

Limitations of this study include the animal model and the species specificity of HCMV that complicates challenge studies. Although rabbits are commonly applied in preclinical vaccine studies with multiple advantages^51–56^, the immune system of rabbits may not represent human B and T cell repertoire. Specifically, the kinetics of T cell response, including initiation, peak immunogenicity, and the memory T cell formation timing, have never been reported. Although we observed a major variation in T cell response at week 6, this timepoint might not be sufficient for rabbits to elicit a mature T cell response for detection. Further, the small animal numbers in each group make it less possible to detect the subtle differences from the vaccine-elicited polyclonal immunity, although multiple mRNA vaccination studies included 6 or fewer in each group to compare the immunogenicity^24,47^.

Overall, our study demonstrates that the multivalent gB mRNA–LNP vaccines did not increase the breadth of the gB-specific humoral and T cell response. This question of multi- vs single-valent anti-viral vaccines has become highly relevant in the design and rollout of the next-generation SARS-CoV-2 mRNA–LNP vaccines to include the Omicron variant. A recent study showed that the SARS-CoV-2 mRNA–LNP vaccine boost in naïve individuals elicited an increased plasma-neutralizing antibody response but a variable antibody response breadth compared to the convalescent individuals^57^. Since the inclusion of the Omicron variant provided minimal gains in protective efficacy against new SARS-CoV-2 strains, their impact on the SARS-CoV-2 pandemic remains unknown^58–61^. While the B cell response breadth against HCMV gB did not benefit from multivalent vaccines in these studies, a higher magnitude T cell response was elicited against monovalent vaccine-mismatched gB genotype, implying that the multivalent HCMV glycoprotein immunization should be considered as a strategy to enhance T cell response breadth. With the ease of including multiple antigens in future mRNA–LNP vaccines, studies should continue to assess the ideal diversity and quantity of antigens that can be administered concurrently in a vaccine to achieve the most effective immunity for HCMV and other pathogens.

## Methods

### gB mRNA production and formulation in lipid nanoparticles

The modified mRNAs encoding HCMV gBs were produced as previously described using T7 RNA polymerase (MEGAscript; Ambion) on codon-optimized linearized plasmids^62,63^. The gB sequences are below gB-1 from Towne strain (GenBank accession number: ACM48044.1; gB-2 from AD169 strain, GenBank accession number: DAA00160.1), gB-3 (Toledo strain, GenBank accession number: ADD39116.1), gB-4 (C194 strain, GenBank accession number: AAA45925), and gB-5 (saliva isolate, GenBank accession number: AZB53144). The mRNAs were transcribed to contain 101 nucleotide-long poly(A) tails. To generate modified nucleoside-containing mRNA, m1Ψ-5’-triphosphate (TriLink) was used instead of UTP. The mRNAs were then capped using an m7G capping kit with 2’-O-methyltransferase (CellScript). Th gB-1 mRNA was purified by fast protein liquid chromatography (FPLC; Akta purifier; GE Healthcare)^64^, and the gB-2, gB-3, gB-4, and gB-5 mRNAs were purified by cellulose-based purification^65^. All mRNAs were analyzed by electrophoresis using agarose gels and stored at −20 °C. The purified m1Ψ-containing HCMV gB mRNAs were encapsulated in LNPs using a self-assembly process. This process involved a rapid mixture of mRNA in pH 4.0 aqueous solution and lipids dissolved in ethanol solution. The LNPs used in this study were similar in composition to those described previously^66,67^. These LNPs contain an ionizable cationic lipid (proprietary to Acuitas), phosphatidylcholine, cholesterol, and polyethylene glycol-lipid. The proprietary lipid and LNP composition are described in US patent US10,221,127. They had a diameter of ~80 nm, as measured by dynamic light scattering using a Zetasizer Nano ZS (Malvern Instruments Ltd.) instrument.

#### Ethics statement

The in vivo immunization and sample collection experiments in rabbits were approved by IACUC of Duke University Medical Center. All experimentation adhered to the USDA Animal Welfare Regulations, PHS Policy on Humane Care and Use of Laboratory Animals, the NIH/NRC Guide for the Care and Use of Laboratory Animals, Assessment and Accreditation of Laboratory Animal Care International (AAALAC) accreditation guidelines, and Duke University Institutional Animal Care and Use Committee (DUIACUC) care and use policies.

### Animal handling, immunization, sample collection, and processing

Adult New Zealand White rabbits (9–10 months old) were a kind gift from Dr. Herman Staats (Duke University) and housed at Duke University. New Zealand White rabbits were applied in this study due to their intermediate size and longer life span, which allowed for a large collection of blood samples and tissue biopsies from a single animal, investigation of the durability of vaccines^55^, and has been validated to recapitulate Fc-mediated effector antibody responses mediated in primates^47^. Rabbits were previously utilized to evaluate the nasal immunogenicity of an unrelated antigen. A total of 50 µg (~0.025 mg/kg) 1-methylpseudouridine-modified gB mRNA encoding single or multiple genotypes in each vaccine was delivered via 6 intradermal injections on the neck. The 50 µg gB-1 mRNA–LNP was reconstituted with 250 µl Dulbecco’s phosphate-buffered saline (DPBS) and each animal in the monovalent vaccine group was given a total of 6 injections with 8.3 µg gB-1 mRNA–LNP; 25 µg gB-1 and 25 µg gB-3 mRNA–LNP was reconstituted with 125 µl DPBS, respectively, and each animal in the bivalent vaccine group was given 3 injections with 8.3 µg gB-1 mRNA–LNP and 3 injections with 8.3 µg gB-3 mRNA–LNP; 10 µg gB-1, 10 µg gB-2, 10 µg gB-3, 10 µg gB-4, and 10 µg gB-5 was reconstituted in 40 µl DPBS, respectively, and each animal in the pentavalent vaccine group was given 1 injection with each genotype and DPBS, respectively. For blood collections from the central ear artery, animals were first sedated with 1 mg/kg of body-weight acepromazine subcutaneously, and the ears were applied with 1% lidocaine topically. The blood was collected via auricular venipuncture and stored in an EDTA-anticoagulated tube before sample processing. Rabbit plasma was separated from whole blood by centrifugation and stored at −80 °C prior to running assays. Peripheral blood mononuclear cells (PBMCs) were isolated by density gradient centrifugation using a lymphocyte–mammal cell separation medium (Cedarlane Laboratories).

For necropsy, the animals were euthanized using 0.5 ml of subcutaneously injected xylazine (100 mg/ml) plus ketamine (500 mg/ml), mixed in a 1:5 ratio, followed by 0.5 ml of intracardiac pentobarbital sodium plus phenytoin sodium (Euthasol). Blood, spleen, and bone marrow samples were collected at necropsy. The collection of 50 ml rabbit blood was obtained from a cardiac puncture with a 21-gauge needle. Splenocytes were isolated from the spleen by manual tissue disruption and crushing through a 100 µm cell strainer, followed by density gradient centrifugation using a Lymphocyte-Rabbit cell separation medium (Cedarlane Laboratories). Bone marrow samples were collected from femurs and flushed through a 100 µm cell strainer.

### Cell culture

Human epithelial kidney 293 T cells (ATCC) were maintained in Dulbecco’s modified Eagle medium (DMEM) supplemented with 10% fetal bovine serum (FBS), 25 mM HEPES, and Pen–Strep. Human retinal pigment epithelial (ARPE-19) cells (ATCC) were maintained in DMEM supplemented with 10% FBS and Pen–Strep. Human foreskin fibroblast HFF-1 cells (ATCC) were maintained in DMEM supplemented with 20% FBS, 2 mM l-glutamine, 25 mM HEPES, Pen–Strep, and 50 µg/ml Gentamicin. Human monocytes THP-1 cells (ATCC) were maintained in Roswell Park Memorial Institute Medium (RPMI) 1640 media supplemented with 10% fetal bovine serum (FBS). Expi293F™ cells (Thermo Fisher Scientific) were cultured in Expi293F™ expression medium (Thermo Fisher Scientific).

#### Protein production

The histidine (His)-tagged soluble gB ectodomain plasmids were designed by removing the transmembrane and cytosolic domains of gB sequences for five genotypes, respectively, and cloned into pcDNA3.1(+) mammalian expression vector (Invitrogen). The gB sequences of the five genotypes are below: gB-1 from Towne strain (GenBank accession number: ACM48044.1; gB-2 from AD169 strain, GenBank accession number: DAA00160.1), gB-3 (Toledo strain, GenBank accession number: ADD39116.1), gB-4 (C194 strain, GenBank accession number: AAA45925), and gB-5 (saliva isolate, GenBank accession number: AZB53144). Expi293F™ cells were transiently transfected with His-tagged gB ectodomain plasmids for five days and later harvested with Nickel-NTA resin (Thermo Fisher Scientific).

The protein production and purification of gB Domain I, Domain II, and Domain I + II was previously reported^4^. In brief, sequences encoding HCMV Merlin strain gB Domain I (codon 133-343), Domain II (codon 112–133, 343–438), and gB Domain I + II (codon 112–438) were tagged with hemagglutinin (HA) tag at the 5’ end, Domain I and Domain I + II were also tagged with an avidin and poly-his tag at the 3’ end. Since gB Domain II was discontinuous, the two segments within gB Domain II were joined by the flexible linker. These gB domain sequences were cloned into pcDNA3.1(+) mammalian expression vector (Invitrogen) and transiently transfected into Expi293F™ cells for five days. The gB Domain I and Domain I + II were purified using Nickel-NTA resin (Thermo Fisher Scientific), and the gB Domain II was purified with lectin resin (VWR).

### Rabbit plasma IgG binding to soluble gB proteins and mAb binding specificity to gB domains by ELISA

To estimate the rabbit plasma IgG binding to soluble gB proteins, 384-well clear-bottom ELISA plates (Corning) were coated overnight at 4 C with 15 ng full-length ΔTM gB and gB ectodomains encoding five genotypes in 0.1 M Carbonate Buffer. The coating plates were washed once with the plate washer after overnight incubation and blocked with assay diluent (1× PBS containing 4% whey, 15% normal goat serum, and 0.5% Tween 20) at room temperature for 1 h. Rabbit plasma was 3-fold serial diluted from 1:100 and then added to the plate for 1 h incubation at room temperature. Cytomegalovirus immune globulin intravenous CytoGam (CSL Behring Healthcare)^68^ was 4-fold serial diluted from 1:1000 and included as assay positive control and plate-to-plate control. The bound rabbit plasma IgG was detected with horseradish peroxidase (HRP)-conjugated polyclonal mouse anti-rabbit IgG (Southern Biotech), and the CytoGam was detected with an HRP-conjugated polyclonal goat anti-human IgG (Southern Biotech) by 1 h incubation at room temperature. The plate was later developed with the SureBlue Reserve tetramethylbenzidine (TMB) peroxidase substrate (KPL) and read at 405 nm. The 50% effective dose end dilution (ED50) was calculated as the plasma dilution that resulted in a 50% reduction of the IgG binding, determined by the method of Reed and Muench^69^.

The mAb binding domain specificity was determined following the similar procedure described above. In total, 384-well clear-bottom ELISA plates (Corning) were coated with 150 ng gB AD-1 domain (MyBiosource), AD-2 domain site 1 (Life Technologies Corporation), Domain I, Domain II, Domain I + II in 0.1 M Carbonate Buffer. The coating plates were washed once with the plate washer after overnight incubation and blocked with assay diluent at room temperature for 1 h. 2M7 mAb was 4-fold serial diluted from 100 µg/ml and then added to the plate for 2-h incubation at room temperature. CytoGam (CSL Behring Healthcare), SM-5, SM-10, and TRL-345 were included as assay positive control (Supl. Fig. 7A–F). CytoGam was 4-fold serial diluted from 500 µg/ml, while SM-5, SM-10, and TRL-345 were 4-fold serial diluted from 100 µg/ml. 2M7 mAb isolated from rabbit PBMCs was detected with an HRP-conjugated polyclonal mouse anti-rabbit IgG (Southern Biotech), and all the positive controls were detected with an HRP-conjugated polyclonal goat anti-human IgG (Southern Biotech) by 1 h incubation at room temperature. The plate was later developed with the SureBlue Reserve tetramethylbenzidine (TMB) peroxidase substrate (KPL) and read at 405 nm. The 50% effective dose end dilution (ED50) was calculated as the plasma dilution that resulted in a 50% reduction of the IgG binding, determined by the method of Reed and Muench^69^.

### gB transfected cell binding assay and gB mRNA–LNP antigenicity

HEK293T cells (ATCC) were cultured overnight to reach ~50% confluency in a T75 flask. For the gB transfected cell binding assay, cells were transfected with 2000 ng gB-T2A-GFP plasmids encoding five gB genotypes, respectively, using the Effectene Transfection Reagent Kit (Qiagen). T2A peptide, composed of 18–22 amino acids, was found in the foot-and-mouth disease virus and is commonly known for its self-cleavage ability^70,71^. Benefiting from the self-cleavage ability of the T2A peptide, the GFP structure would not modify the gB structure on the cell surface after transfection. The GFP expression was measured to determine the transfection efficiency of gB-T2A-GFP plasmids. For antigenicity assay, cells were co-transfected with gB mRNA–LNP encoding each genotype and GFP plasmid as previously described^22^. Transfected cells were incubated at 37 °C, 5% CO_2_ for 48 h, and then detached with 1× PBS. Cells were re-suspended in DMEM complete media and counted using Countess Automated Cell Counter (Invitrogen). 200,000 cells were plated in a 96-well round bottom plate and blocked with 1:1000 Human TruStain FcX™ (Biolegend) for 10 minutes. After blocking, the cells were washed with 1× PBS once and centrifuged at 1200×*g* for 5 min to aspirate the washing buffer. Rabbit plasma was diluted 1:100, and CytoGam was diluted 1:5000 in DMEM complete media, included as positive control and plate-to-plate control. After rabbit plasma incubation with transfected cells at 37 °C for 2 h, cells were washed with 1× PBS + 1% FBS and centrifuged at 1200×*g* for 5 min twice. Dead HEK293T cells were prepared by heating at 95 °C for 5 min as a dead cell control for the following live/dead staining procedure. The stained cells were washed with 1× PBS + 1% FBS and centrifuged at 1200×*g* for 5 min twice and stained with the 1:1000 Far Red live/dead staining (Invitrogen) at room temperature for 20 min. After washing with 1× PBS + 1% FBS and centrifuged at 1200×*g* for 5 min twice, cells were then incubated with PE-conjugated secondary antibody at 4 °C for 25 min. The secondary antibody for the rabbit plasma is PE-conjugated polyclonal mouse anti-rabbit IgG (Southern Biotech), and that for the CytoGam is mouse anti-human IgG (Southern Biotech). The stained cells were washed with 1× PBS + 1% FBS twice and fixed with 1× PBS + 4% formalin at room temperature for 15 min. After fixation, the fixed cells were washed with 1× PBS + 1% FBS twice and resuspended in 100 µl PBS for the acquisition. Events were acquired on an LSR-II flow cytometer (BD Biosciences) using the HTS (high throughput screening) cassette. Data was analyzed with Flowjo software (Tree Star, Inc.). GFP expression was measured as the transfection efficiency. As the (Supl. Fig. 3A) shows, the average transfection efficiency of gB-T2A-GFP plasmids is between 34 and 49% depending on the gB genotype (gB1: 39%; gB2: 34%; gB-3: 42%; gB-4: 44%; gB-5: 49%). The IgG binding to cell-associated gB was measured by the % of GFP + PE+ population. Non-specific binding of PE-conjugated mouse anti-rabbit IgG Fc and mouse anti-human IgG Fc was corrected in the analysis. Due to the variations among the transfection efficiency of gB-T2A-GFP plasmids, the IgG binding of week 6, 10, and 30 samples was normalized by GFP expression MFI of week 0 for comparison.

### Glycoprotein B peptide microarray

The rabbit plasma IgG binding to linear 15-mer gB peptides was assessed as previously described^4^. We designed the 15-mer peptides, overlapping by 10 residues, that covered the amino acids 1–77 of the gB open reading frame from Towne (gB-1), AD169r (gB-2), and Toledo (gB-3) strain since the most variable region lies in the codon 26–70^16–18^. A total of 27 peptides were printed in triplicate on the PepStar multi-well array (JPT Peptide). Following the protocol from JPT Peptide, the peptide microarray slide was fixed in the 96-well chamber cassette. The blocking buffer is 3% BSA (Bovine serum albumin) in 1× Tris-buffered saline (TBS) + 0.1% Tween20 buffer, and the wash buffer is 1× TBS + 0.1% Tween20. Rabbit plasma was centrifuged at 45,000 rpm for 5 min and diluted 1:250 in the blocking buffer. After adding the diluted plasma, the slide was wrapped in aluminum foil and placed on the rotator for 1-h incubation at 150 rpm, 30 °C. The slide was then washed with 150 µl washing buffer four times and incubated with 1:500 Alexa Fluor® 647 AffiniPure Goat Anti-Rabbit IgG (H + L) (Jackson Immuno Research) for 1-h incubation at 150 rpm, 30 °C. The stained slide was washed with 150 µl washing buffer four times again and further washed with 150 µl DI water twice before drying by centrifuge spin at 1350 rpm for 5 min. The dried microarray slide was scanned at 635 nm by Genepix 4100 A and analyzed by GenePix Pro 6.1 software (Molecular Devices). The plasma binding to linear 15-mer gB peptides was calculated by (feature intensity-background intensity) at 635 nm, and the median peptide binding of each of the triplicates was reported. The cutoff for positive peptide binding was defined by the average binding of the secondary non-specific antibody binding, Alexa Fluor® 647 AffiniPure Goat Anti-Rabbit IgG (H + L), plus two standard deviations.

### Neutralization

The fibroblast neutralization titer was measured on HFF-1 cells (ATCC), and the epithelial neutralization titer was detected on ARPE-19 cells (ATCC). Five thousand cells/well HFF-1 cells or ARPE-19 cells were resuspended in complete DMEM media + 20% FBS or complete DMEM media + 10% FBS, respectively. HFF-1 cells or ARPE cells were later plated in 384-well black and clear-bottom plates (Corning) overnight at 37 °C. Rabbit plasma was heat-inactivated at 65 °C for 1 h to inactivate the proteins that impact the neutralization activity (e.g., complement). Next, rabbit plasma was 3-fold serial diluted from 1:8, and CytoGam was 3-fold serial diluted from 1:80, included as assay positive control and plate-to-plate control. The diluted rabbit plasma was incubated with Towne, AD169r, or Toledo virus (a kind gift from Dr. Ravit Boger), respectively, at 37 °C, 5% CO_2_ for 1 h. To assess the rabbit plasma neutralization antibody response with the effect of complement, purified rabbit complement (Cedarlane Laboratories) was mixed in the complete DMEM media+20% FBS at a ratio of 1:4. After 1-h incubation, the mixture of rabbit plasma and HCMV was added in duplicate to wells containing HFF-1 or ARPE-19 cells for a further incubation. HFF-1 cells were incubated at 37 °C, 5% CO_2_ for another 20–24 h, while ARPE-19 cells were further incubated at 37 °C for 44–48 h. Infected cells were then fixed with 1× PBS + 4% formalin at room temperature for 15 min before staining. The staining buffer is 1× DPBS + 1% FBS + 0.3% Triton X-100. Fixed cells were stained with 1:1000 mouse anti-HCMV IE-1 monoclonal antibody (MAB810; Millipore) for 1 h and then stained with 1:1000 goat anti-mouse IgG-AF488 (Millipore) for another 1 h at room temperature. The nuclear stain was later performed by incubating DAPI (4 = ,6-diamidino-2-phenylindole) nuclear stain for 10 min at room temperature. After staining, the cells were resuspended in 1× PBS before detection. The total cells and AF488+ cells were counted on a Cellomics ArrayScan reader (Thermo Fisher Scientific) or ImageXpress Pico Automated Cell Imaging System (Molecular Devices). The % infection rate was determined by the AF488+ cells/total cells. Neutralization titers (ID50) were calculated based on a 50% reduction of the % infected cells via the method of Reed and Muench^69^.

### ADCP of whole HCMV virions

Towne, AD169, and Toledo virus were conjugated with DMSO-dissolved AF647–N-hydroxysuccinimide ester (Invitrogen) with constant agitation at room temperature for 1 h. The conjugation reaction was quenched with 1 M Tris-HCl, pH 8.0. In a 96-well round-shaped plate, rabbit plasma was diluted 1:30 in 1× PBS + 1% FBS. CytoGam (CSL Behring Healthcare) was 3-fold serial diluted from 1:30 and as positive control and plate-to-plate control, while PBS was included as negative control. The diluted sera and controls were incubated with viruses at 37 °C for 2 h. After 2-h incubation, 250,000 cells/ml THP-1 cells (ATCC) were resuspended in RPMI + 10% FBS media, and 200 µl THP-1 cells were added to the sera-virus mixture. The 96-well plate was centrifuged at 1200×*g*, 4 °C for 1 h to allow spinoculation and then incubated at 37 °C, 5% CO_2_, for an additional 1 hour. The cells were spun down at 1200×*g* for 5 min and washed with 1× PBS + 1% FBS once before staining with Aqua Live/Dead stain (Invitrogen) at room temperature for 25 min. The stained cells were washed with 1× PBS + 1% FBS twice and fixed with 1× PBS + 4% formalin at room temperature for 15 min. After fixation, the fixed cells were washed with 1× PBS + 1% FBS twice and resuspended in 100 µl PBS for the acquisition. Events were acquired on an LSR-II flow cytometer (BD Biosciences) using the HTS (high throughput screening) cassette. Data were analyzed with Flowjo software (Tree Star, Inc.). The ADCP activity was determined by the AF647+ cells from the live cell population using the PBS negative control as the threshold (Supl. Fig. 5A, B).

### Monoclonal antibody screening and isolation

Rabbit memory B cell culture followed by mAb screening and cloning was previously reported^40,72^. The week 10 rabbit PBMCs from the monovalent and pentavalent vaccine groups (around 1E + 07 cells per sample, 1 animal from each group) were mixed to ensure the cell number was enough for the following cell culture. A total of 600 IgG+ memory B cells were incubated and enriched with HCMV AD169r strain, then seeded into 384-well plates, at a density of one cell/per well with pre-coated EL4-B5 feeder cells (Kerafast). The feeder cells were irradiated with 50 Gy in a gamma radiation chamber before use. The single memory B cells were cultured in complete RPMI 1640 medium supplemented with IL-2 (10 U/ml, R&D Systems), IL-21 (10 U/ml, R&D Systems), at 37 °C, 5% CO_2_, 93% humidity for 14 days. The B-cell culture supernatants were collected. These supernatants were tested in post-fusion full-length gB binding ELISA and AD169r neutralization assay to screen the positive cell candidates. The total RNA from the cell candidates was isolated with RNeasy Micro Kit (Qiagen) and converted to cDNA using iScript™ cDNA Synthesis Kit (Bio-Rad). The IgG heavy and light chain genes were amplified by PCR as previously described^40^. The plasmids encoding the heavy and light chain genes were later transiently transfected in Expi293F cells (Thermo Fisher Scientific), and the recombinant mAbs were purified by Protein A/G affinity chromatography as reported^40^.

### gB-specific T cell IFN-γ ELISpot assay

Rabbit IFN-γ ELISpot BASIC kit (MabTech) was used to determine IFN-γ production from rabbit PBMCs and splenocytes. Following the protocol from MabTech, the 96-well MultiScreenHTS IP Filter Plate (Millipore) was activated with 35% ethanol and coated with 15 µg/ml MT327 coating antibody overnight at 4 °C. On the following day, the 96-well plate was washed with sterile PBS five times and blocked with RPMI + 10% FBS media for 30 min at room temperature. Frozen rabbit primary PBMCs and splenocytes were thawed in RPMI + 10% FBS media and counted by Countess™ 3 Automated Cell Counter (Invitrogen). 200,000 primary rabbit cells were plated in each well and stimulated with PepMix™ HCMVA UL55 peptides (JPT Peptide Technologies) or customized overlapping 15-mer peptide pools targeting gB-3 and gB-5 variable regions (Genscript, Supl. Table 2) overnight at 37 °C, 5% CO_2_. Cells were also stimulated with 0.4 µg/ml PMA (MilliporeSigma) and 4 µg/ml ionomycin (Sigma-Aldrich) for positive control and DMSO (Sigma-Aldrich) or RPMI + 10% FBS media only for negative control. After 20-h incubation, cells were removed, and the plate was washed with sterile PBS five times before staining. The staining buffer is 1× DPBS + 0.5% FBS. The secreted cytokine was incubated with 0.1 µg/ml MT318-biotin detection antibody in the staining buffer for 2 h at room temperature, followed by a five-time sterile PBS wash. The plate was later incubated with 1:1000 Streptavidin-ALP conjugate in staining buffer for 1 h at room temperature, followed by a five-time sterile PBS wash. An alkaline phosphate conjugate substrate kit (Bio-Rad) was applied for substrate color development. The plate was developed with color development solution for 25 min at room temperature, and the reaction was stopped by incubating with 0.5% Tween-20 in 1× DPBS for 10 min. After removing the color development solution, the plate was washed with tap water four times and dried overnight before reading using ImmunoSpot® S6 Ultimate M2 Analyzer (Cellular Technology Limited). The IFN-γ-positive spots were imaged and counted using ImmunoSpot® Software (Cellular Technology Limited).

### Statistical analysis

In the longitudinal analysis of full-length gB IgG Elisa, we utilized a non-parametric repeated measures test due to the small sample with permutations used due to a singular covariance matrix^73,74^. For the TCB and ectodomain analysis, a multivariate Kruskal–Wallis test was used with 100000 permutations to conduct inference using the coin package in R^75^. For the neutralizing and ADCP analysis, Kruskal–Wallis was again used-with permutations due to the small sample size at each time point and virus^75^. For all analyses, multiple testing correction was done using FDR^76^ with significance at an FDR-adjusted *p*-value less than 0.1 and an unadjusted *p*-value less than 0.05. This was with the exception of when only adjusted for two tests in which a Bonferroni correction was used. For any post-hoc analyses (within specific viruses), multiple testing correction was done using the FWER controlling Holm procedure^73^. Secondary tests between individual vaccines (Monovalent vs. Bivalent/Pentavalent combined, for example) were done using the Wilcoxon Rank Sum test in the coin package^75^. All analyses were done within R^77^. For the ELISPOT T cell response analysis, we performed Kruskal–Wallis as above with multiple testing corrections via Bonferroni.

### Reporting summary

Further information on research design is available in the Nature Research Reporting Summary linked to this article.

### Supplementary information

Supplementary figures and tables

Dataset for main figures

Dataset for supplementary figures

REPORTING SUMMARY

## Data Availability

All data generated or analyzed during this study are included in this article and its supplementary files. The data used for figure generation and statistical analysis can also be found at https://github.com/HsuanYuanWang/Rabbit-gB-mRNA-multivalent-vaccine.git.
