# Supplementary material for: Multivalent cytomegalovirus glycoprotein B nucleoside modified mRNA vaccines did not demonstrate a greater antibody breadth
Source: NPJ Vaccines. 2024 Feb 20;9:38. doi: 10.1038/s41541-024-00821-3 (PMC10879498; doi:10.1038/s41541-024-00821-3)
Supplement: Supplementary file 1 — Supplementary figures and tables [file 41541_2024_821_MOESM1_ESM.pdf]

# Sup Fig. 1

## A

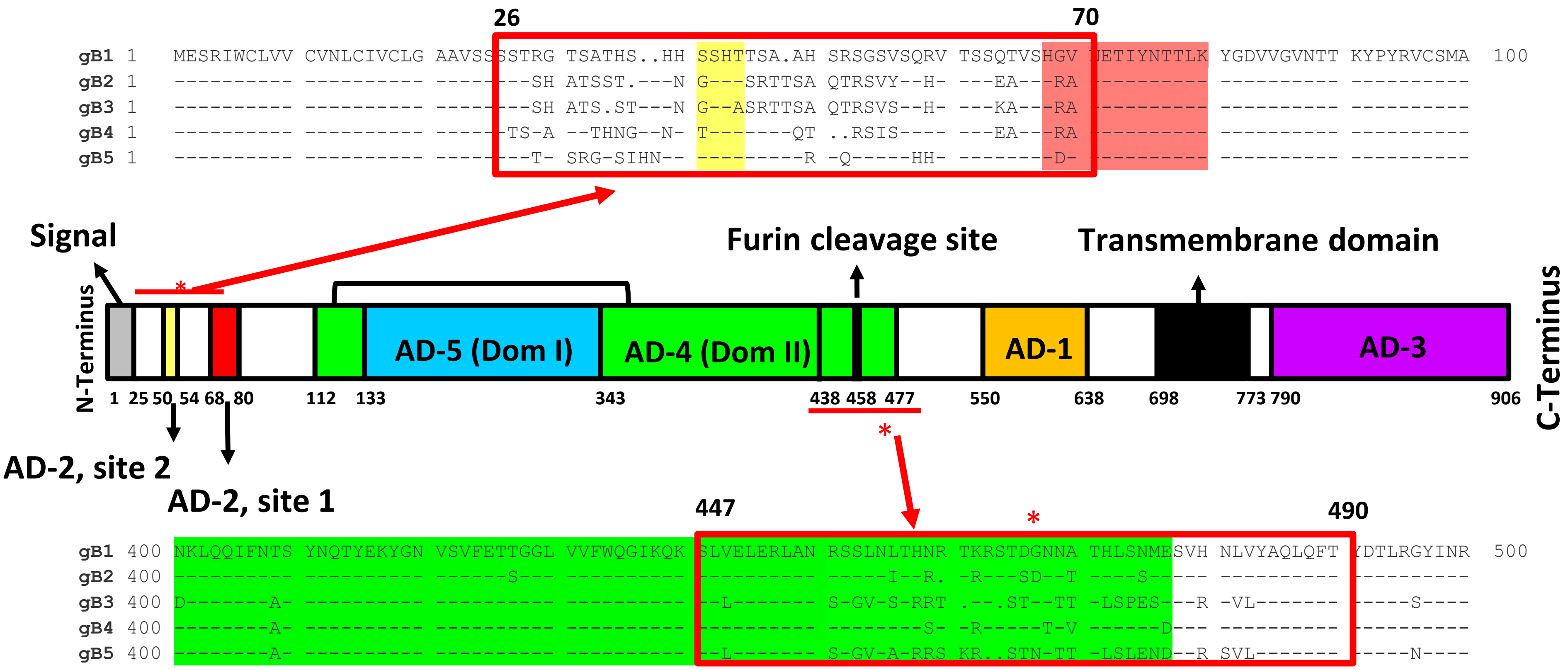


## B

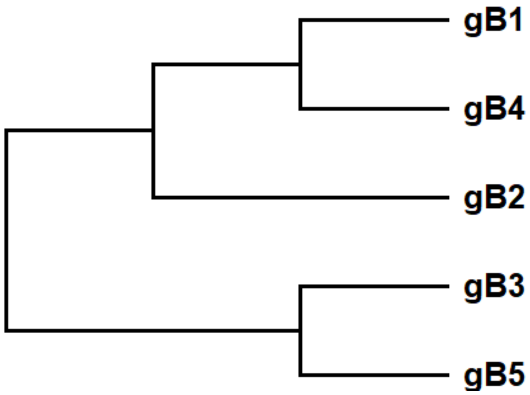


| gB Genotypes Sequence Alignment (Percent Identify Matrix) | | | | | |
|---|---|---|---|---|---|
| | **gB5** | **gB3** | **gB2** | **gB1** | **gB4** |
| **gB5** | 100 | 93.36 | 92.14 | 93.04 | 91.14 |
| **gB3** | 93.36 | 100 | 95.35 | 93.03 | 93.46 |
| **gB2** | 92.14 | 95.35 | 100 | 95.47 | 95.24 |
| **gB1** | 93.04 | 93.03 | 95.47 | 100 | 96.13 |
| **gB4** | 91.14 | 93.46 | 95.24 | 96.13 | 100 |

**C**

```
gB1 MESRIWCLVV CVNLCIVCLG AAVSSSSTRG TSATHS..HH SSHTTSA.AH SRSGSVSQRV TSSQTVSHGV NETIYNTTLK YGDVVGVNTT KYPYRVCSMA  100
gB2 ---------- ---------- --------SH ATSST.---N G---SRTTSA QTRSVY--H- ---EA---RA ---------- ---------- ----------
gB3 ---------- ---------- --------SH ATS.ST---N G--ASRTTSA QTRSVS--H- ---KA---RA ---------- ---------- ----------
gB4 ---------- ---------- ------TS-A --THNG--N- T-------QT ..RSIS---- ---EA---RA ---------- ---------- ----------
gB5 ---------- ---------- --------T- SRG-SIHN-- ---------R -Q-----HH- --------D- ---------- ---------- ----------

gB1 QGTDLIRFER NIVCTSMKPI NEDLDEGIMV VYKRNIVAHT FKVRVYQKVL TFRRSYAYIH TTYLLGSNTE YVAPPMWEIH HINSHSQCYS SYSRVIAGTV  200
gB2 ---------- --I------- ---------- ---------- ---------- ---------Y ---------- ---------- ---KFA---- ------G---
gB3 ---------- -----P---- ---------- ---------- ---------- ---------- ---------- ---------- ---------- ----------
gB4 --------D- -----P---- ---------- ---------- ---------- ---------- ---------- ---------- ---------- ----------
gB5 ---------- -----PV--- ---------- ---------- ---------- ---------Y S--------- ---------- ---KFA---- ---------L

gB1 FVAYHRDSYE NKTMQLMPDD YSNTHSTRYV TVKDQWHSRG STWLYRETCN LNCMVTITTA RSKYPYHFFA TSTGDVVDIS PFYNGTNRNA SYFGENADKF  300
gB2 ---------- ------I--- ---------- ---------- ---------- ----L----- ---------- -------Y-- ---------- ----------
gB3 ---------- -------L-- ------I--- ---------- ---------- ---------- ---------- ---------- ---------T ----------
gB4 ---------- ---------- ---------- ---------- ---------- ---------- ---------- ---------- ---------- ----------
gB5 ---------- -------S-- ---------- ---------- -----H---- ------M-A- ---------- ---------- ---------T ----------

gB1 FIFPNYTIVS DFGRPNSALE THRLVAFLER ADSVISWDIQ DEKNVTCQLT FWEASERTIR SEAEDSYHFS SAKMTATFLS KKQEVNMSDS ALDCVRDEAI  400
gB2 ---------- ------A-P- ---------- ---------- ---------- ---------- ---------- ---------- ---------- ----------
gB3 ---------- ----A---P- ---------- ---------- ---------- ---------- ---------- ---------- ---------P V-----NQ-L
gB4 ---------- ---------- ---------- ---------- ---------- ---------- ----E----- ---------- ---------- ---------L
gB5 ---------- ------A-Q- ---------- ---------- ---------- ---------- ---K------ ---------- ---------P V------Q-L

gB1 NKLQQIFNTS YNQTYEKYGN VSVFETTGGL VVFWQGIKQK SLVELERLAN RSSLNLTHNR TKRSTDGNNA THLSNMESVH NLVYAQLQFT YDTLRGYINR  500
gB2 ---------- ---------- ------S--- ---------- ---------- -----I--R. -R---SD--T ----S----- ---------- ----------
gB3 D-------A- ---------- ---------- ---------- --L------- S-GV-S-RRT .--.ST--TT -LSPES---R -VL------- -----S----
gB4 --------A- ---------- ---------- ---------- ---------- --------S- -R-----T-V ------D--- ---------- ----------
gB5 --------A- ---------- ---------- ---------- --L------- S-GV-A-RRS KR..STN-TT -LSLEND--R SVL------- -----N----

gB1 ALAQIAEAWC VDQRRTLEVF KELSKINPSA ILSAIYNKPI AARFMGDVLG LASCVTINQT SVKVLRDMNV KESPGRCYSR PVVIFNFANS SYVQYGQLGE  600
gB2 ---------- ---------- ---------- ---------- ---------- ---------- ---------- ---------- ---------- ----------
gB3 ---------- ---------- ---------- ---------- ---------- ---------- ---------- ---------- -------V-- ----------
gB4 --T------- ---------- ---------- ---------- ---------- ---------- ---------- ---------- ---------- ----------
gB5 ---------- ---------- ---------- ---------- ---------- ---------- --------TI -D-T------ -----T---- -H--------

gB1 DNEILLGNHR TEECQLPSLK IFIAGNSAYE YVDYLFKRMI DLSSISTVDS MIALDIDPLE NTDFRVLELY SQKELRSINV FDLEEIMREF NSYKQRVKYV  700
gB2 ---------- ---------- ---------- ---------- ---------- ---------- ---------- -------S-- ---------- ----------
gB3 ---------- -----F---- ---------- ---------- ---------- ---------- ---------- -------S-- ---------- ----------
gB4 ---------- ---------- ---------- ---------- ---------- ---------- ---------- -------S-- ---------- ----------
gB5 ---------- ---------- ---------- ---------- ---------- ---------- ---------- -------S-- ---------- ----------

gB1 EDKVVDPLPP YLKGLDDLMS GLGAAGKAVG VAIGAVGGAV ASVVEGVATF LKNPFGAFTI ILVAIAVVII IYLIYTRQRR LCMQPLQNLF PYLVSADGTT  800
gB2 ---------- ---------- ---------- ---------- ---------- ---------- ---------- T--------- --T------- ----------
gB3 ---------- ---------- ---------- ---------- ---------- ---------- ---------- ---------- ---------- ----------
gB4 ---------- ---------- ---------- ---------- ---------- ---------- ---------- ---------- ---------- ----------
gB5 ---------- ---------- ---------- ---------- ---------- ---------- ---------- T--------- --T------- ----------

gB1 VTSGNTKDTS LQAPPSYEES VYNSGRKGPG PPSSDASTAA PPYTNEQAYQ MLLALVRLDA EQRAQQNGTD SLDGQTGTQD KGQKPNLLDR LRHRKNGYRH  900
gB2 ----S----- ---------- ---------- ---------- ---------- -----A---- ---------- ---------- ---------- ----------
gB3 ---------- ---------- ---------- ---------- ---------- -----A---- ---------- ---------- ---------- ----------
gB4 ---------- ---------- ---------- ---------- ---------- ---------- ---------- ---------- ---------- ----------
gB5 ----S----- ---------- ---------- ---------- ---------- -----A---- ---------- ---------- ---------- ----------

gB1 LKDSDEEENV  910
gB2 ----------
gB3 ----------
gB4 ----------
gB5 ----------
```

**Sup Figure 1. Schematic representation of HCMV gB sequence with antigenic domains and the most variable regions among 5 gB genotypes.**

(A) The full opening reading frame of HCMV gB is shown from the N-terminus (left) to the C-terminus (right). The gB sequence consists of ectodomain, transmembrane domain (black), and the cytosolic domain. The AD-1 (orange), AD-2 site 1 (red), AD-2 site 2 (yellow), AD-4 (also known as Dom II, green), and AD-5 (also known as Dom I, blue) are in ectodomain, while AD-3 (purple) belongs to cytosolic domain. This figure was adapted from Burke et al. [1], Chandramouli et al. [2], and Nelson et al. [3]. The most variable regions defining the five gB genotypes are codons 26-70 and codons 441-490. These regions were aligned using Clustal W and Clustal X version 2.0 [4] and presented by SeqPublish. The five gB sequences included in the vaccine design are: gB-1 (C327A strain, M60929.1), gB-2 (C336A strain, M60931.1), gB-3 (BE/37/2011 strain, KP745723.1), gB-4 (C194A strain, M60926.2), and gB-5 (17_saliva_7-24-2003 isolate, MK157431.1).

(B) Phylogenetic tree of full gB open reading frame encoding 5 gB genotypes. The phylogenetic analysis was performed following the bootstrap method with 100 replications by Molecular Evolutionary Genetics Analysis version 11 (Tamura 2011). Clades representing 5 individual gB genotypes are color-coded : gB-1, black circle; gB-2, red square; gB-3, green upward triangle; gB-4, blue diamond; gB-5, orange downward triangle. The precent identity matrix comparing five gB genotypes was generated using Clustal W and Clustal X version 2.0 (94).

(C) The complete sequence alignment of the five HCMV gB genotypes using Clustal W and Clustal X version 2.0 (94) and presented by SeqPublish. The five gB sequences included in the vaccine design are: gB-1 (C327A strain, M60929.1), gB-2 (C336A strain, M60931.1), gB-3 (BE/37/2011 strain, KP745723.1), gB-4 (C194A strain, M60926.2), and gB-5 (17_saliva_7-24-2003 isolate, MK157431.1).

# Sup Fig. 2

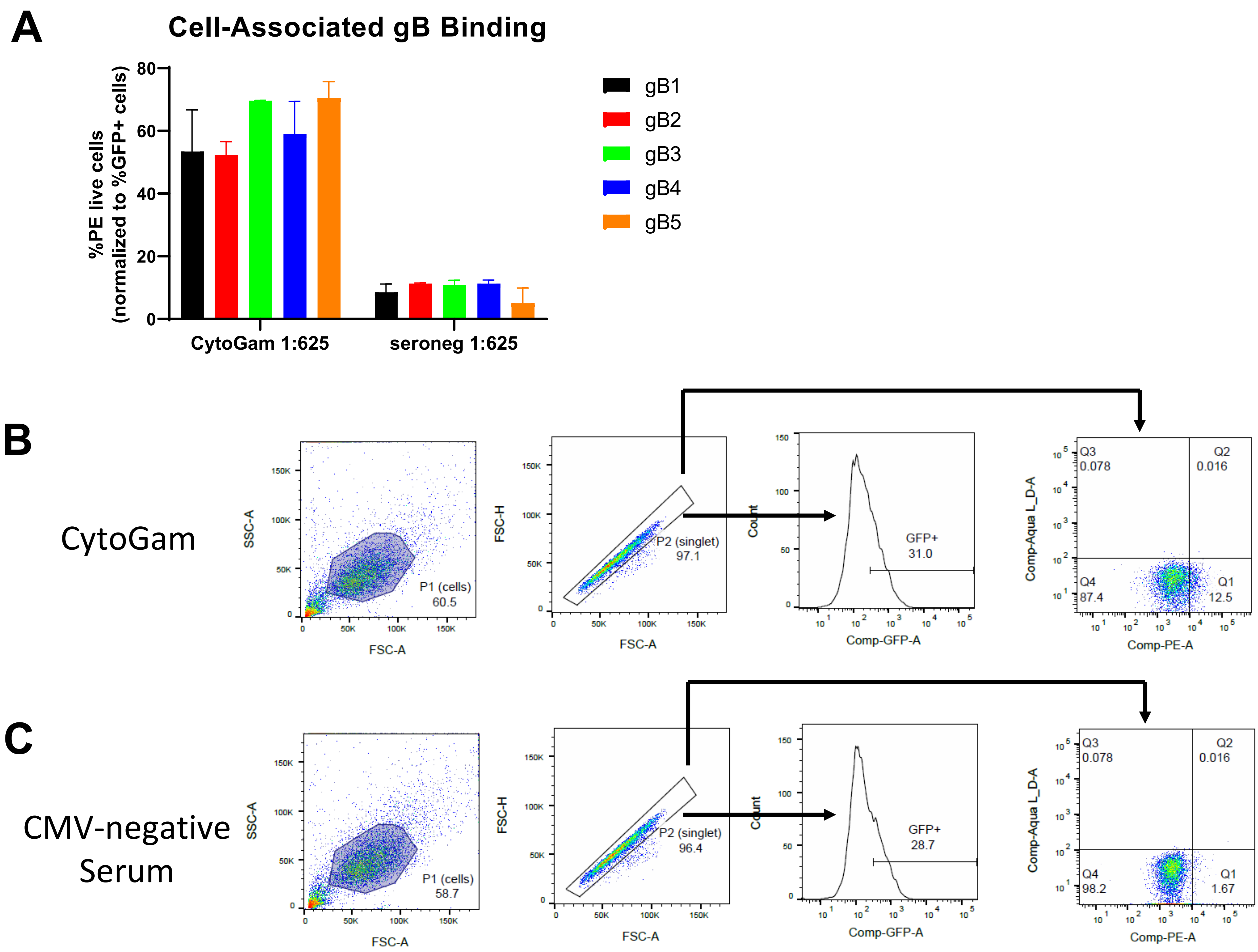
A
Cell-Associated gB Binding
%PE live cells (normalized to %GFP+ cells)
80
60
40
20
0
CytoGam 1:625
seroneg 1:625
gB1
gB2
gB3
gB4
gB5
B
CytoGam
SSC-A
FSC-A
P1 (cells)
60.5
FSC-H
P2 (singlet)
97.1
Count
Comp-GFP-A
GFP+
31.0
Comp-Aqua L_D-A
Comp-PE-A
Q3
0.078
Q2
0.016
Q4
87.4
Q1
12.5
C
CMV-negative Serum
P1 (cells)
58.7
P2 (singlet)
96.4
GFP+
28.7
Q3
0.078
Q2
0.016
Q4
98.2
Q1
1.67

**Sup Fig. 2 mRNA-LNPs encoding gB genotype antigens encoding each genotype similar antigenicity.**

(A) Transfection efficiency of gB mRNA-LNPs encoding each genotype *in vitro*. mRNA-LNPs encoding each gB genotype were co-transfected with GFP DNA plasmid in HEK 293 T cells *in vitro* to compare the antigenicity of each gB antigen. The transfected cells were incubated with HCMV immune globulin (CytoGam) or seronegative plasma to measure the polyclonal IgG binding to cell-associated gBs. Each data bar represents the average % PE+ live cell population normalized to % GFP+ population in duplicate, with error bar indicating the standard error of mean. Polyclonal IgG binding to gB genotypes are color-coded: gB-1 (black), gB-2 (red), gB-3 (green), gB-4 (blue), and gB-5 (orange). No statistical analysis was performed due to the small sample size.
(B-C) Gating strategy of gB antigenicity for CytoGam (B) and HCMV-seronegative plasma (C). The 293T cells were determined by SSC-A and FSC-A, designated as P1 population. The P2 singlet population was later obtained by gating the P1 population through FSC-A and FSC-H. Next, the singlet P2 population was gated by GFP+ population based on the mock transfection control and live PE+ population (Q1) based on the HCMV-seronegative plasma, respectively. The % PE+ live population shown in Q1 was normalized to GFP+ population to eliminate the transfection efficiency effect.

Sup Fig. 3

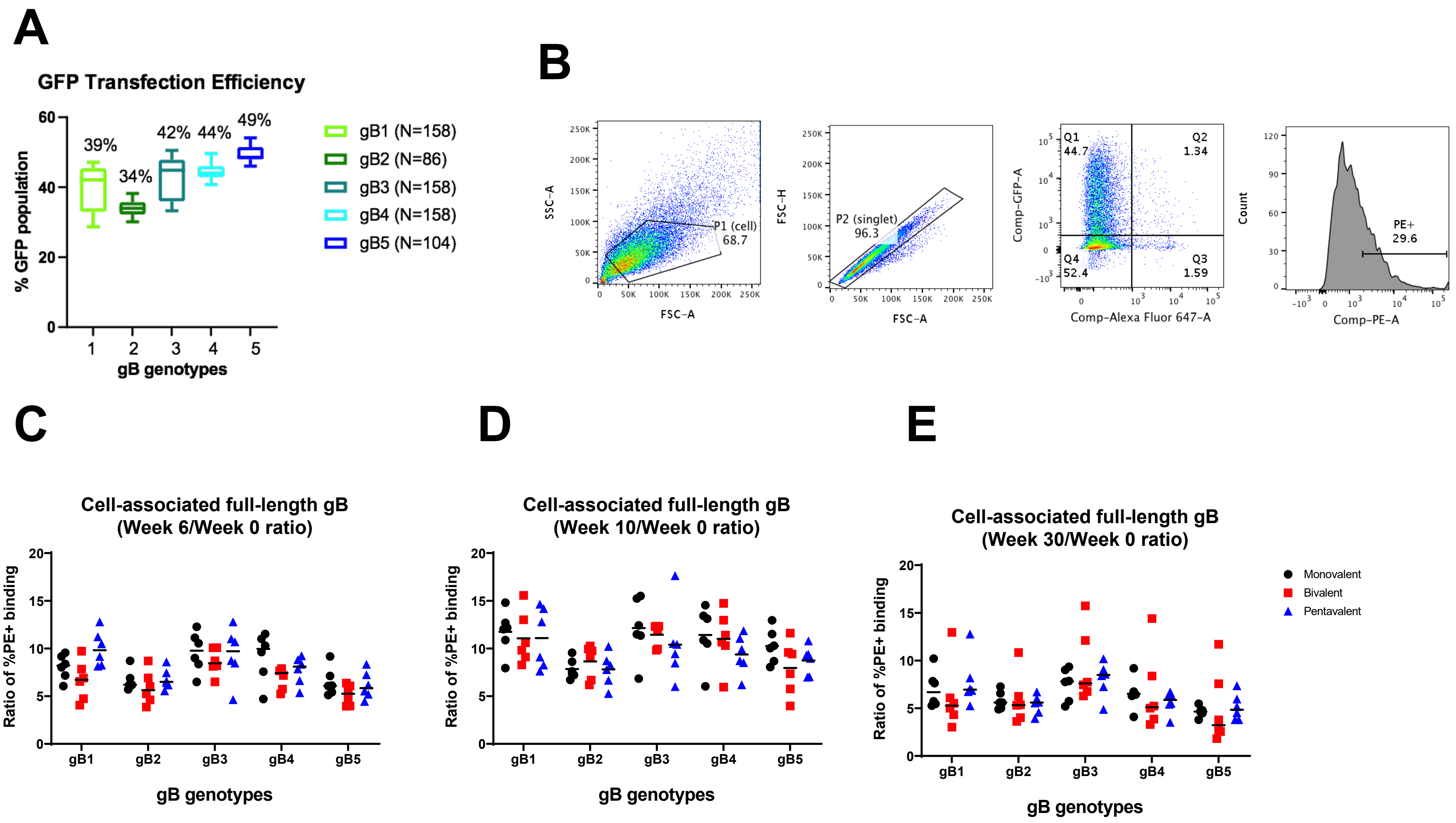
A
GFP Transfection Efficiency
% GFP population
gB genotypes
39%
34%
42%
44%
49%
gB1 (N=158)
gB2 (N=86)
gB3 (N=158)
gB4 (N=158)
gB5 (N=104)
B
P1 (cell)
68.7
P2 (singlet)
96.3
Q1
44.7
Q2
1.34
Q4
52.4
Q3
1.59
PE+
29.6
SSC-A
FSC-A
FSC-H
Comp-GFP-A
Comp-Alexa Fluor 647-A
Count
Comp-PE-A
C
Cell-associated full-length gB
(Week 6/Week 0 ratio)
D
Cell-associated full-length gB
(Week 10/Week 0 ratio)
E
Cell-associated full-length gB
(Week 30/Week 0 ratio)
Ratio of %PE+ binding
gB genotypes
gB1
gB2
gB3
gB4
gB5
Monovalent
Bivalent
Pentavalent

**Sup Fig. 3 Monovalent, bivalent, and pentavalent gB mRNA-LNP vaccinated rabbit plasma IgG elicited a similar binding breadth to cell-associated gB of 5 genotypes.**

(A) The transfection efficiency of gB-T2A-GFP plasmids encoding 5 genotypes, respectively, was determined by %GFP expression. The %GFP expression was shown by box and whisker plot, with the mean value labeled on top. These values were color-coded based on the gB genotypes: gB-1, lawn green; gB-2, Forest green; gB-3, Paris green; gB-4, sky blue; gB-5, blue. (B) Gating strategy of gB transfected cell binding assay. The 293T cells were determined by SSC-A and FSC-A, designated as P1 population. The P2 singlet population was later obtained by gating the P1 population through FSC-A and FSC-H. Next, the live GFP+ P2 population was shown in Q1, and the %PE+ population was compared from the Q1 population. (C-E) Rabbit plasma IgG binding breadth to full-length gB encoding 5 genotypes at week 6 (C), week 10 (D), and week 30 (E) by gB-transfected cell binding assay. Data points are shown as the IgG binding response normalized to that of the week 0 samples to eliminate the discrepancies among the transfection efficiency. Each data point represents the normalized IgG binding response of one individual animal, with the median response labeled by a black line. Black circles: rabbits immunized with monovalent vaccine; red squares: those immunized with bivalent vaccine; blue triangles: those immunized with pentavalent vaccine.

# Sup Fig. 4

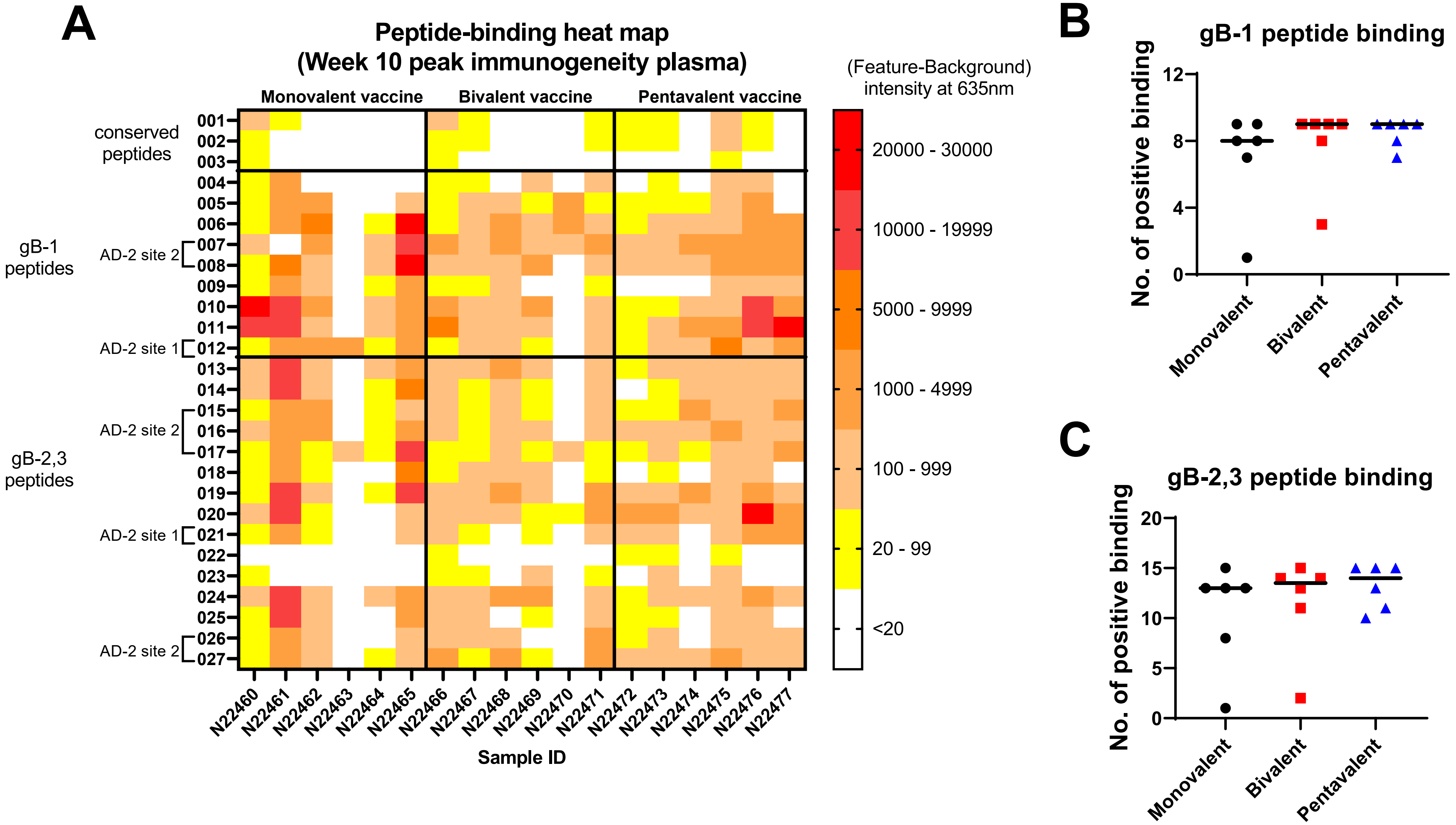
A
Peptide-binding heat map
(Week 10 peak immunogeneity plasma)
Monovalent vaccine
Bivalent vaccine
Pentavalent vaccine
conserved peptides
gB-1 peptides
gB-2,3 peptides
AD-2 site 2
AD-2 site 1
001
002
003
004
005
006
007
008
009
010
011
012
013
014
015
016
017
018
019
020
021
022
023
024
025
026
027
N22460
N22461
N22462
N22463
N22464
N22465
N22466
N22467
N22468
N22469
N22470
N22471
N22472
N22473
N22474
N22475
N22476
N22477
Sample ID
(Feature-Background) intensity at 635nm
20000 - 30000
10000 - 19999
5000 - 9999
1000 - 4999
100 - 999
20 - 99
<20
B
gB-1 peptide binding
No. of positive binding
0
4
8
12
Monovalent
Bivalent
Pentavalent
C
gB-2,3 peptide binding
No. of positive binding
0
5
10
15
20
Monovalent
Bivalent
Pentavalent

**Sup Fig. 4 Monovalent, bivalent, and pentavalent gB mRNA-LNP vaccinated rabbit plasma IgG at week 10 did not show a statistical different binding pattern to linear gB peptides of gB-1 or gB-2/3 genotypes.**

(A) The breadth of rabbit plasma IgG binding to 15-mer linear gB peptides covering the amino acid codons 1-77 of gB-1 or gB2/3 genotypes (27 unique peptides) at peak immunogenicity (week 10), measured by peptide microarray. Each row indicates a linear gB peptide: 001-003, conserved peptides; 004-012, gB-1 peptides; 013-027, gB2/3 peptides. Each column is one animal from each vaccine group. The plasma IgG binding to linear 15-mer gB peptides was calculated by (feature-background) intensity at 635nm, and the average peptide median binding (feature-background) intensity at 635nm of each of the triplicates was reported. (B-C) The positive binding MFI cutoff was defined by the average binding of the secondary non-specific antibody binding plus two standard deviations. Each data point represents the number of peptides bound by plasma IgG from one individual animal, with the median response labeled by a black line. Black circles: rabbits immunized with monovalent vaccine; red squares: those immunized with bivalent vaccine; blue triangles: those immunized with pentavalent vaccine.

# Sup Fig. 5

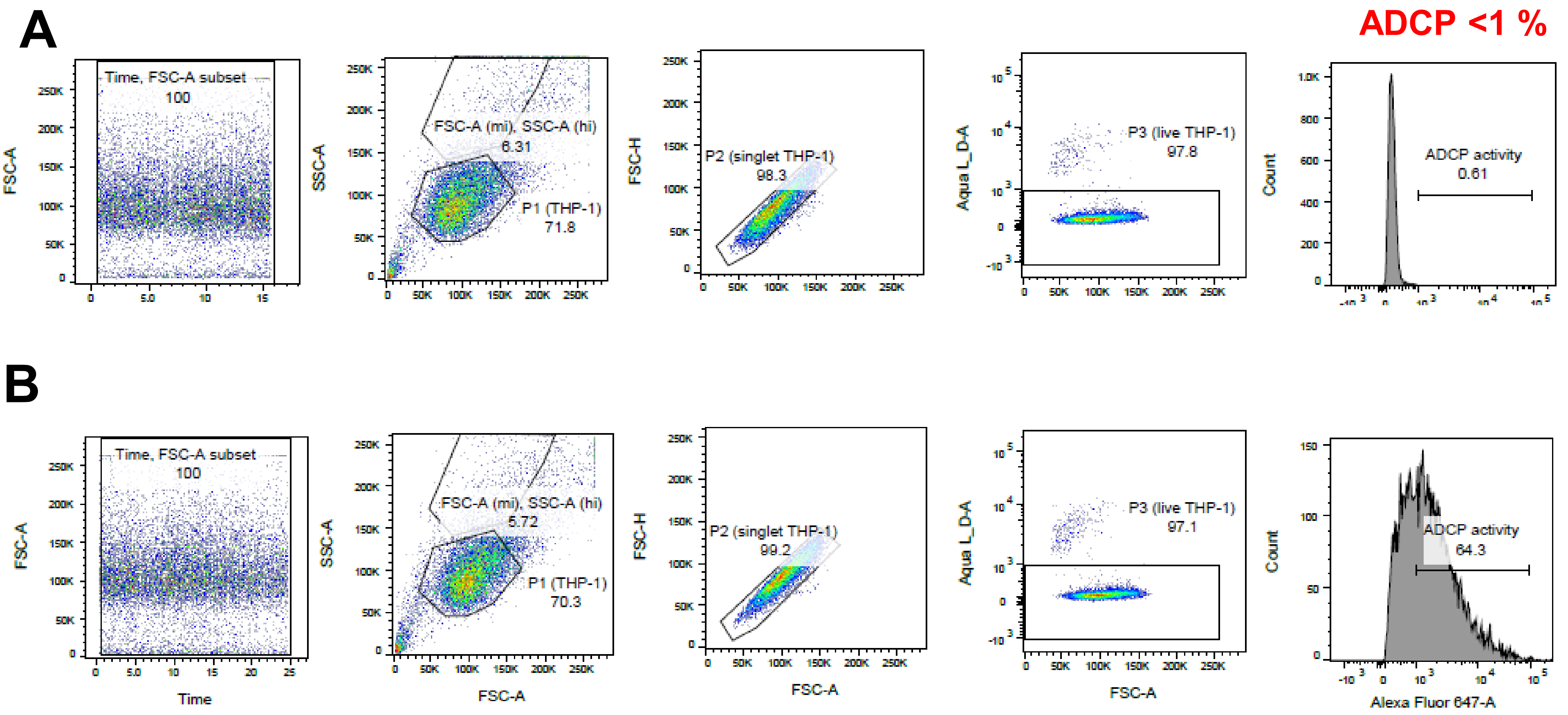


**Sup Fig. 5 Gating strategy of ADCP for negative control (PBS) and positive control (CytoGam)**

(A-B) The THP-1 cells were first gated for acquisition time and the cells were determined by SSC-A and FSC-A, designated as P1 population. The P2 singlet THP-1 population was later obtained by gating the P1 population through FSC-A and FSC-H. Next, the live THP-1 cell population was by aqua L/D stain as P3, and the % ADCP activity was determined by the AF647+ cells from the live cell population using the PBS negative control as the threshold. ADCP activity from PBS negative control (A) and CytoGam positive control (B).

# Sup Fig. 6

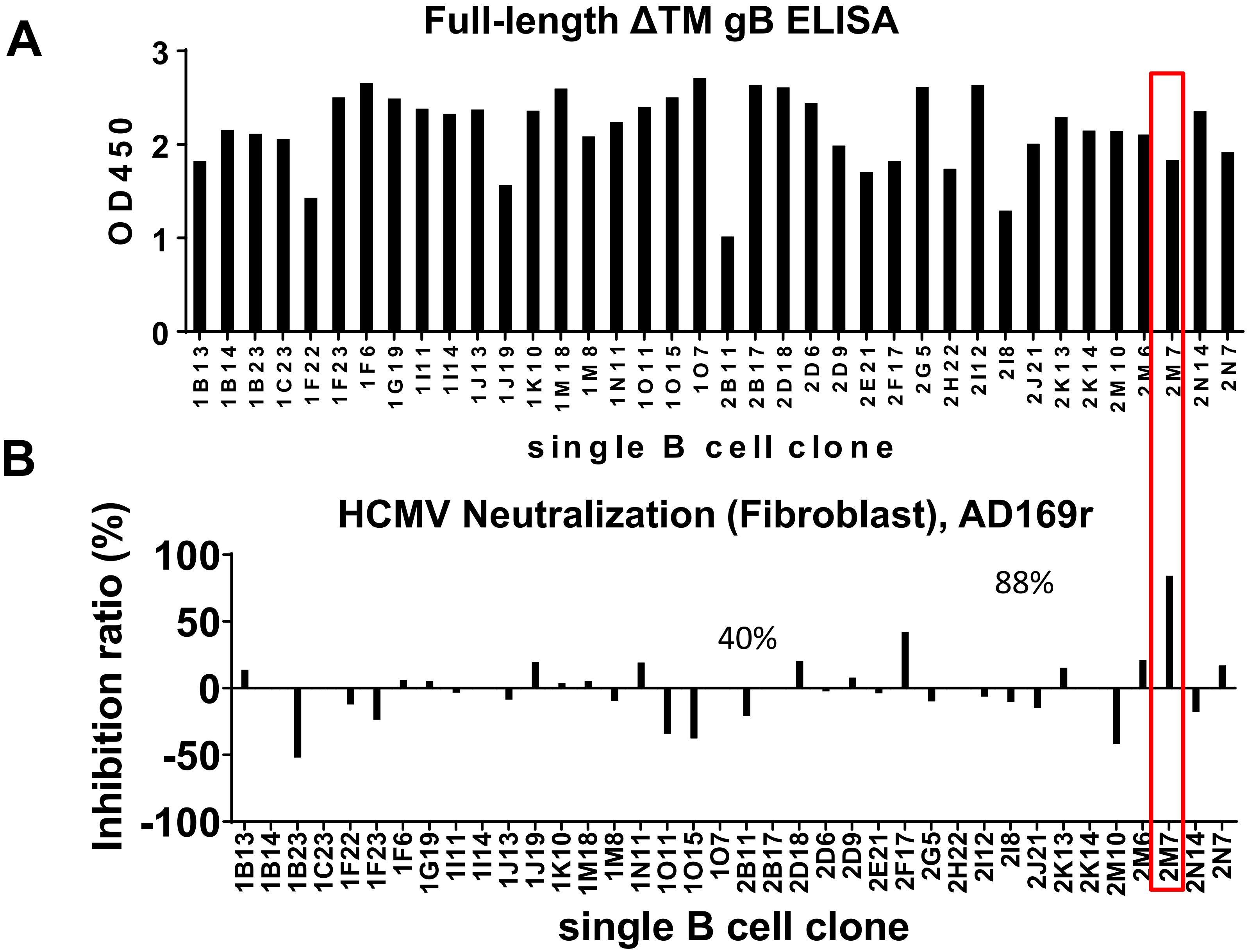


**Sup Fig. 6 Monoclonal antibody screening from memory B cell clones.**

(A) Among 600 single memory B cell clones, the supernatant (1:20 dilution) of 38 clones demonstrated a binding response to post-fusion full-length gB by ELISA. (B) The fibroblast neutralization potency of the supernatants from the 38 gB-binding clones (1:20 dilution) was examined by neutralization assay against HCMV AD169r strain. Among 38 mAbs, only 2M7 showed the strongest neutralization potency.

# Sup Fig. 7

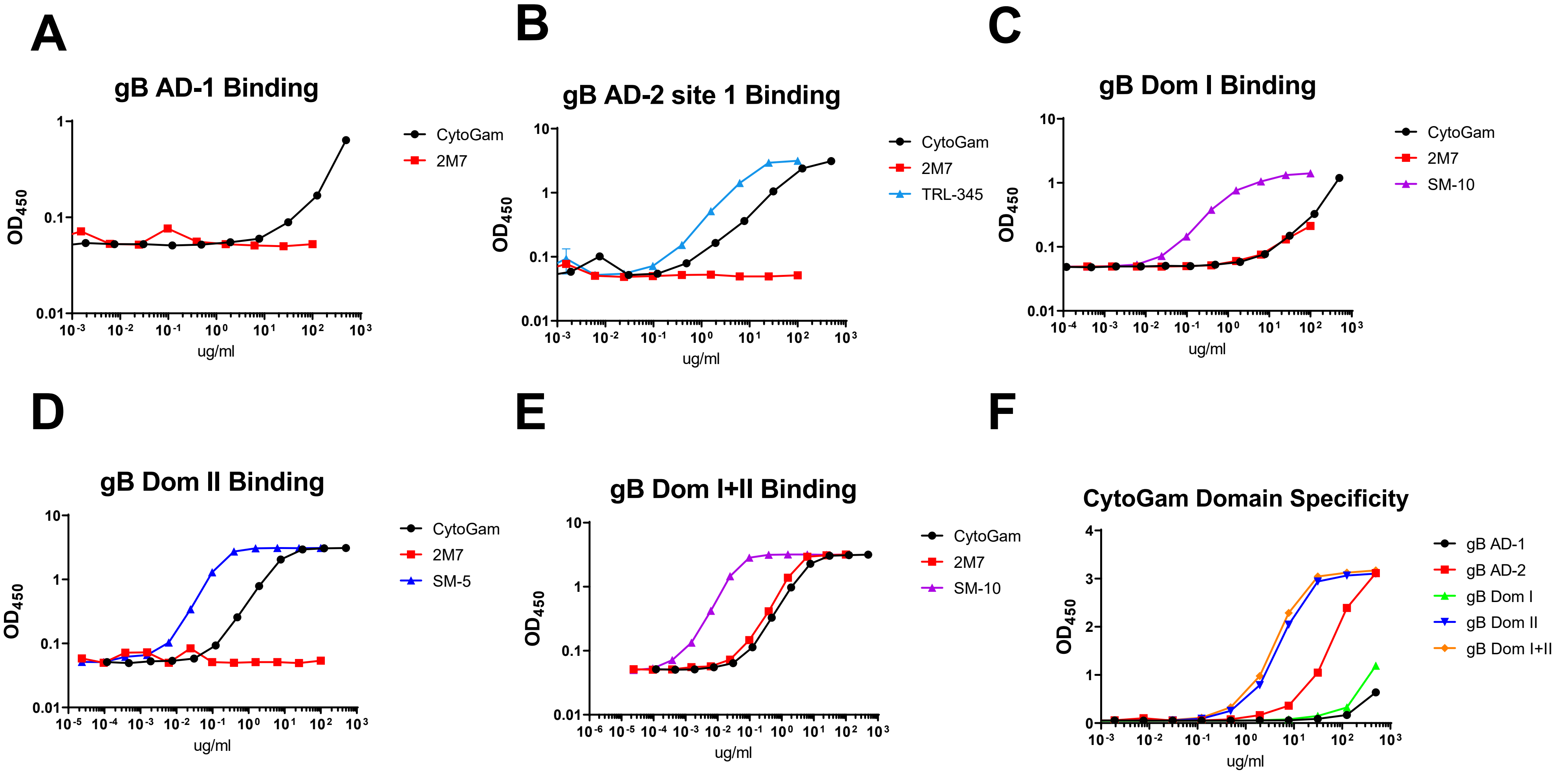


**Sup Fig. 7 Positive controls of gB domain specificity confirmed 2M7 only showed binding to gB Dom I+II.**

(A-E) The gB domain specificity of 2M7 was determined by comparing binding to gB AD-1 (A), AD-2 (B), Domain I (C), Domain II (D), and domain I+II (E). The positive controls for each antigen are CytoGam (A-F, black circle), TRL-345 (B, light blue triangle), SM-10 (C, E, purple triangle), and SM-5 (D, blue triangle). (F) The gB domain specificity of CytoGam, measured by ELISA. CytoGam binding to gB domains is color-coded with symbols: black circle, AD-1; red square, AD-2 site 1; green upward triangle, Domain I; blue downward triangle, Domain II; orange diamond, Domain I+II.

# Sup Fig. 8

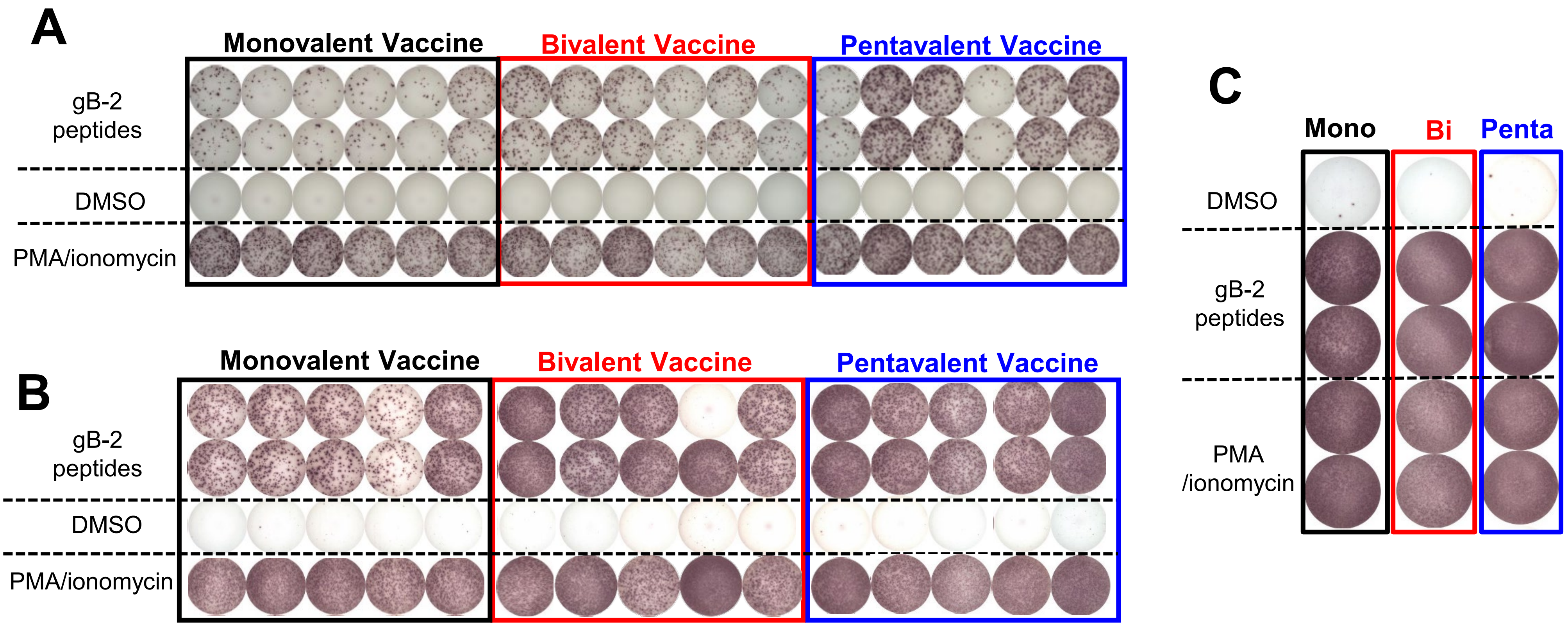


**Sup Fig. 8 Representative wells of IFN-r+ cells stimulated with gB-2 peptides, DMSO, or PMA/ionomycin from rabbit PBMCs at week 6, and splenocytes at week 30 or 43.**

gB-2-specific IFN-r+ cells from rabbit PBMCs or splenocytes were measured by ELISPOT. gB-2 peptide pool stimulation were performed in duplicate. Black rectangle: rabbits immunized with monovalent vaccine; red rectangle: those immunized with bivalent vaccine; blue rectangle: those immunized with pentavalent vaccine. Representative wells of rabbit PBMCs at week 6 (A), splenocytes at week 30 (B) and week 43 (C). (B) One well from the bivalent vaccine group incubated with gB-2 peptides did not show any binding and thus was removed for analysis. (C) 1 rabbit from each vaccine group received another booster at week 41 and underwent necropsy at week 43.

# Sup Fig. 9

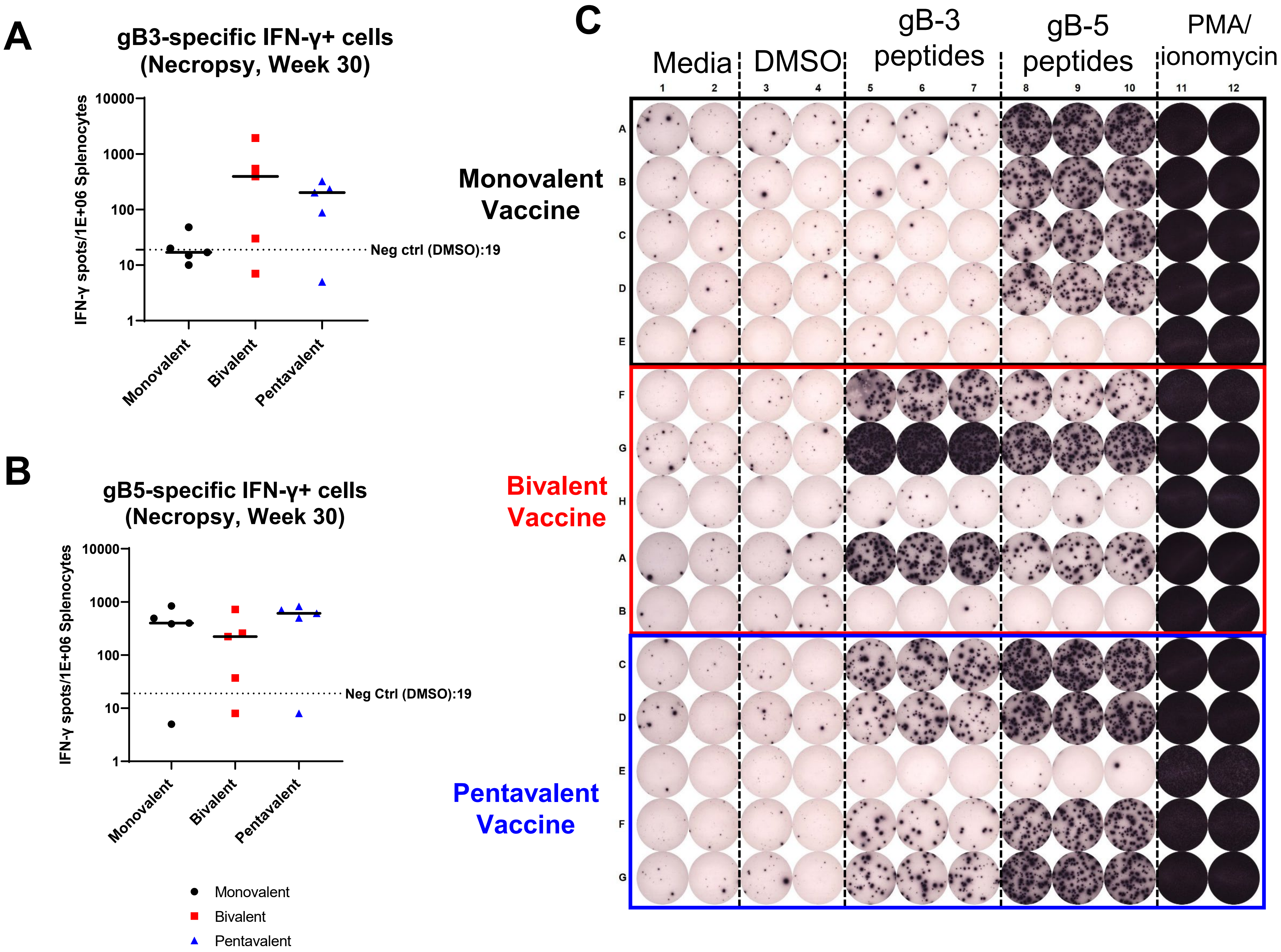
A
gB3-specific IFN-γ+ cells
(Necropsy, Week 30)
IFN-γ spots/1E+06 Splenocytes
10000
1000
100
10
1
Neg ctrl (DMSO):19
Monovalent
Bivalent
Pentavalent
B
gB5-specific IFN-γ+ cells
(Necropsy, Week 30)
IFN-γ spots/1E+06 Splenocytes
10000
1000
100
10
1
Neg Ctrl (DMSO):19
Monovalent
Bivalent
Pentavalent
Monovalent
Bivalent
Pentavalent
C
Media
DMSO
gB-3 peptides
gB-5 peptides
PMA/ ionomycin
1 2 3 4 5 6 7 8 9 10 11 12
A
B
C
D
E
F
G
H
A
B
C
D
E
F
G
Monovalent Vaccine
Bivalent Vaccine
Pentavalent Vaccine

**Sup Fig. 9 Multivalent gB mRNA-LNP vaccine elicited a stronger gB-3 (Toledo strain) but not gB-5-specific IFN-r+ cell response in splenocytes at necropsy.**

gB-3 and gB-5-specific IFN-r+ cells from rabbit splenocytes were measured by ELISPOT. Overlapping 15-mer peptide libraries targeting gB-3 and gB-5 variable regions, respectively, were incubated with rabbit splenocytes in triplicate.
(A) Quantification of IFN-r+ spots from rabbit splenocytes stimulated with gB-3 variable regions peptides at necropsy (week 30) from monovalent, bivalent, and pentavalent vaccine groups.
(B) Quantification of IFN-r+ spots from rabbit splenocytes stimulated with gB-5 variable regions peptides at necropsy (week 30) from monovalent, bivalent, and pentavalent vaccine groups. 15 animals underwent necropsy at week 30, while 3 animals (1 animal from each group) underwent necropsy at week 43 due to an extra boost prior to B cell isolation at week 41. Each data point represents one peptide-stimulated well from individual animals, with the lines designating the median. Black circles: rabbits immunized with monovalent vaccine; red squares: those immunized with bivalent vaccine; blue triangles: those immunized with pentavalent vaccine. Kruskal-Wallis test with Bonferroni correction was performed for any overall diference. The assay limitation was determined by the average spot count of the cells incubated with DMSO only (negative control).
(C) Wells of IFN-r+ cells stimulated with gB-3 or gB-5 variable region peptides (in triplicate), DMSO (in duplicate), media (in duplicate) or PMA/ionomycin (in duplicate) from rabbit splenocytes at week 30. Black rectangle: rabbits immunized with monovalent vaccine; red rectangle: those immunized with bivalent vaccine; blue rectangle: those immunized with pentavalent vaccine.

# Sup Table 1. Linear overlapping 15-mer gB peptide sequence.

| Peptide No. | gB peptide sequence | gB genotype | Peptide No. | gB peptide sequence | gB genotype |
|---|---|---|---|---|---|
| 001 | MESRIWCLVVCVNLC | 1,2,3,4,5 | 015 | SSTSHATSSTHNGSH | 2,3 |
| 002 | WCLVVCVNLCIVCLG | 1,2,3,4,5 | 016 | ATSSTHNGSHTSRTT | 2,3 |
| 003 | CVNLCIVCLGAAVSS | 1,2,3,4,5 | 017 | HNGSHTSRTTSAQTR | 2,3 |
| 004 | IVCLGAAVSSSSTRG | 1 | 018 | TSRTTSAQTRSVSSQ | 2,3 |
| 005 | AAVSSSSTRGTSATH | 1 | 019 | SAQTRSVSSQHVTSS | 2,3 |
| 006 | SSTRGTSATHSHHSS | 1 | 020 | SVSSQHVTSSEAVSH | 2,3 |
| 007 | TSATHSHHSSHTTSA | 1 | 021 | HVTSSEAVSHRANET | 2,3 |
| 008 | SHHSSHTTSAAHSRS | 1 | 022 | WCLVVCVNLCYVCLG | 2,3 |
| 009 | HTTSAAHSRSGSVSQ | 1 | 023 | CVNLCYVCLGAVVSS | 2,3 |
| 010 | AHSRSGSVSQRVTSS | 1 | 024 | YVCLGAVVSSSSTSH | 2,3 |
| 011 | GSVSQRVTSSQTVSH | 1 | 025 | AVVSSSSTSHATSSA | 2,3 |
| 012 | RVTSSQTVSHGVNET | 1 | 026 | SSTSHATSSAHNGSH | 2,3 |
| 013 | IVCLGAAVSSSSTSH | 2,3 | 027 | ATSSAHNGSHTSRTT | 2,3 |
| 014 | AAVSSSSTSHATSST | 2,3 | | | |

# Sup Table 2. Overlapping 15-mer peptide libraries targeting gB-3 and gB-5 variable regions.

| Peptide library targeting gB-3 variable regions | | |
|---|---|---|
| **Peptide No.** | **gB-3 peptide sequence** | **Codon** |
| 001 | SSTSHATSSAHNGSH | 26-40 |
| 002 | HATSSAHNGSHTSRT | 30-44 |
| 003 | SAHNGSHTSRTTSAQ | 34-48 |
| 004 | GSHTSRTTSAQTRSV | 38-52 |
| 005 | SRTTSAQTRSVSSQH | 42-56 |
| 006 | SAQTRSVSSQHVTSS | 46-60 |
| 007 | RSVSSQHVTSSEAVS | 50-64 |
| 008 | SQHVTSSEAVSHRAN | 54-68 |
| 009 | LANSSGVNSTRRTKR | 445-459 |
| 010 | SGVNSTRRTKRSTGN | 449-463 |
| 011 | STRRTKRSTGNTTTL | 453-467 |
| 012 | TKRSTGNTTTLSLES | 457-451 |
| 013 | TGNTTTLSLESESVR | 461-455 |
| 014 | TTLSLESESVRNVLY | 464-459 |
| 015 | LESESVRNVLYAQLQ | 468-463 |
| 016 | SVRNVLYAQLQFTYD | 472-467 |

| Peptide library targeting gB-5 variable regions | | |
|---|---|---|
| **Peptide No.** | **gB-5 peptide sequence** | **Codon** |
| 001 | SSTTGSRGTSIHNHH | 26-40 |
| 002 | GSRGTSIHNHHSSHT | 30-44 |
| 003 | TSIHNHHSSHTTSAA | 34-48 |
| 004 | NHHSSHTTSAARSQS | 38-52 |
| 005 | SHTTSAARSQSGSVS | 42-56 |
| 006 | SAARSQSGSVSHHVT | 46-60 |
| 007 | SQSGSVSHHVTSSQT | 50-64 |
| 008 | SVSHHVTSSQTVSHD | 54-68 |
| 009 | HVTSSQTVSHDVNET | 58-72 |
| 010 | LANSSGVNATRRSKR | 447-461 |
| 011 | SGVNATRRSKRSTNN | 451-465 |
| 012 | ATRRSKRSTNNTTTL | 455-469 |
| 013 | SKRSTNNTTTLSLEN | 459-473 |
| 014 | TNNTTTLSLENDSVR | 463-477 |
| 015 | TTLSLENDSVRSVLY | 467-481 |
| 016 | LENDSVRSVLYAQLQ | 471-485 |
| 017 | SVRSVLYAQLQFTYD | 475-489 |

## References for supplementary info:

1. Burke, H. G. & Heldwein, E. E. Crystal Structure of the Human Cytomegalovirus Glycoprotein B. PLoS Pathog 1–21 (2015) doi:10.1371/journal.ppat.1005227.
2. Chandramouli, S. et al. Structure of HCMV glycoprotein B in the postfusion conformation bound to a neutralizing human antibody. Nat Commun 6, 8176 (2015).
3. Nelson, C. S. et al. Human Cytomegalovirus Glycoprotein B Nucleoside-Modified mRNA Vaccine Elicits Antibody Responses with Greater Durability and Breadth than MF59-Adjuvanted gB Protein Immunization. J Virol 94, (2020).
4. Larkin, M. A. et al. Clustal W and Clustal X version 2.0. Bioinformatics 23, 2947–2948 (2007).
