# Supplementary material for: Multivalent cytomegalovirus glycoprotein B nucleoside modified mRNA vaccines did not demonstrate a greater antibody breadth
Source: NPJ Vaccines. 2024 Feb 20;9:38. doi: 10.1038/s41541-024-00821-3 (PMC10879498; doi:10.1038/s41541-024-00821-3)
Supplement: Supplementary file 4 — REPORTING SUMMARY [file 41541_2024_821_MOESM4_ESM.pdf]

Corresponding author(s): Sallie R Permar
Last updated by author(s): Jan 17, 2024

Reporting Summary

Nature Portfolio wishes to improve the reproducibility of the work that we publish. This form provides structure for consistency and transparency in reporting. For further information on Nature Portfolio policies, see our Editorial Policies and the Editorial Policy Checklist.

Statistics

For all statistical analyses, confirm that the following items are present in the figure legend, table legend, main text, or Methods section.

n/a	Confirmed	
☐	☒	The exact sample size (*n*) for each experimental group/condition, given as a discrete number and unit of measurement
☐	☒	A statement on whether measurements were taken from distinct samples or whether the same sample was measured repeatedly
☐	☒	The statistical test(s) used AND whether they are one- or two-sided<br>*Only common tests should be described solely by name; describe more complex techniques in the Methods section.*
☐	☒	A description of all covariates tested
☐	☒	A description of any assumptions or corrections, such as tests of normality and adjustment for multiple comparisons
☐	☒	A full description of the statistical parameters including central tendency (e.g. means) or other basic estimates (e.g. regression coefficient) AND variation (e.g. standard deviation) or associated estimates of uncertainty (e.g. confidence intervals)
☐	☒	For null hypothesis testing, the test statistic (e.g. *F*, *t*, *r*) with confidence intervals, effect sizes, degrees of freedom and *P* value noted<br>*Give P values as exact values whenever suitable.*
☐	☒	For Bayesian analysis, information on the choice of priors and Markov chain Monte Carlo settings
☐	☒	For hierarchical and complex designs, identification of the appropriate level for tests and full reporting of outcomes
☐	☒	Estimates of effect sizes (e.g. Cohen's *d*, Pearson's *r*), indicating how they were calculated

Our web collection on statistics for biologists contains articles on many of the points above.

Software and code

Policy information about availability of computer code

Data collection	Spectramax plate reader (Thermo Scientific), ImageXpress Pico (Molecular Devices), FACSDiva, ImmunoSpot® S6 Ultimate M2 Analyzer (Cellular Technology Limited), Genepix 4100A (Molecular Devices)
Data analysis	Graph Pad Prism, Flow Jo, Excel, R

For manuscripts utilizing custom algorithms or software that are central to the research but not yet described in published literature, software must be made available to editors and reviewers. We strongly encourage code deposition in a community repository (e.g. GitHub). See the Nature Portfolio guidelines for submitting code & software for further information.

Data

Policy information about availability of data

All manuscripts must include a data availability statement. This statement should provide the following information, where applicable:

- Accession codes, unique identifiers, or web links for publicly available datasets
- A description of any restrictions on data availability
- For clinical datasets or third party data, please ensure that the statement adheres to our policy

Data Availability
All data generated or analyzed during this study are included in this article and its supplementary files. The data used for figure generation and statistical analysis can also be found at https://github.com/HsuanYuanWang/Rabbit-gB-mRNA-multivalent-vaccine.git.

Research involving human participants, their data, or biological material

Policy information about studies with human participants or human data. See also policy information about sex, gender (identity/presentation), and sexual orientation and race, ethnicity and racism.

Reporting on sex and gender	*Use the terms sex (biological attribute) and gender (shaped by social and cultural circumstances) carefully in order to avoid confusing both terms. Indicate if findings apply to only one sex or gender; describe whether sex and gender were considered in study design; whether sex and/or gender was determined based on self-reporting or assigned and methods used. Provide in the source data disaggregated sex and gender data, where this information has been collected, and if consent has been obtained for sharing of individual-level data; provide overall numbers in this Reporting Summary. Please state if this information has not been collected. Report sex- and gender-based analyses where performed, justify reasons for lack of sex- and gender-based analysis.*
Reporting on race, ethnicity, or other socially relevant groupings	*Please specify the socially constructed or socially relevant categorization variable(s) used in your manuscript and explain why they were used. Please note that such variables should not be used as proxies for other socially constructed/relevant variables (for example, race or ethnicity should not be used as a proxy for socioeconomic status). Provide clear definitions of the relevant terms used, how they were provided (by the participants/respondents, the researchers, or third parties), and the method(s) used to classify people into the different categories (e.g. self-report, census or administrative data, social media data, etc.) Please provide details about how you controlled for confounding variables in your analyses.*
Population characteristics	*Describe the covariate-relevant population characteristics of the human research participants (e.g. age, genotypic information, past and current diagnosis and treatment categories). If you filled out the behavioural & social sciences study design questions and have nothing to add here, write "See above."*
Recruitment	*Describe how participants were recruited. Outline any potential self-selection bias or other biases that may be present and how these are likely to impact results.*
Ethics oversight	*Identify the organization(s) that approved the study protocol.*

Note that full information on the approval of the study protocol must also be provided in the manuscript.

Field-specific reporting

Please select the one below that is the best fit for your research. If you are not sure, read the appropriate sections before making your selection.

☒ Life sciences ☐ Behavioural & social sciences ☐ Ecological, evolutionary & environmental sciences

For a reference copy of the document with all sections, see nature.com/documents/nr-reporting-summary-flat.pdf

Life sciences study design

All studies must disclose on these points even when the disclosure is negative.

Sample size	We included 6 rabbits each group in our study based on the feasibility, use of this group size in previous literature, and the ability to identify shifts in antibody breadth. We planned our study and determined the primary endpoint was to measure gB genotype-specific antibody binding and neutralizing responses, while the secondary endpoint was to measure gB-specific T cell responses.<br><br>Several vaccination studies using New Zealand White rabbit models have included 6 or less in each group to compare humoral and T cell responses. Pollara et al. (PMID: 30842326) reported HIV vaccine-elicited antibody binding and functional antibody responses (neutralizing antibody response, antibody-dependent phagocytosis, and antibody-dependent cytotoxic response) from rabbits (N=6 each group). Nelson et al. (PMID: 32051265) compared gB-elicited humoral and T cell response from rabbits immunized with HCMV full-length gB or gB ectodomain protein subunit vaccines and nucleoside-modified mRNA-LNP vaccine (N=6 each group). Pardi et al. (PMID: 30974332) characterized the humoral response elicited by rabbits immunized with nucleoside-modified mRNA-LNP vaccine encoding HIV-1 envelope, influenza virus hemagglutinin, or poly(C) (N=5 each group). Based on these studies, we planned 6 animals each group to compare vaccine-elicited humoral and T cell responses.
Data exclusions	One data point in Sup Fig 8(B) was excluded because there were no spots detected in the technical repeat well.
Replication	We run positive controls monitored by Levy-Jennings curves to validate consistency within 3 standard deviation between each experiment. Within each experiment samples are run at minimum in duplicate and depending on the nature of the assay a specific CV is required for data to be included. If replicates are outside of range that data point is repeated.
Randomization	All rabbit plasma samples were allocated randomly to plates for each assay and run at the same time.
Blinding	All rabbit plasma samples were allocated randomly to plates for each assay and run at the same time.

Reporting for specific materials, systems and methods

We require information from authors about some types of materials, experimental systems and methods used in many studies. Here, indicate whether each material, system or method listed is relevant to your study. If you are not sure if a list item applies to your research, read the appropriate section before selecting a response.

Materials & experimental systems

n/a	Involved in the study
☐	☒ Antibodies
☐	☒ Eukaryotic cell lines
☒	☐ Palaeontology and archaeology
☐	☒ Animals and other organisms
☒	☐ Clinical data
☒	☐ Dual use research of concern
☒	☐ Plants

Methods

n/a	Involved in the study
☒	☐ ChIP-seq
☐	☒ Flow cytometry
☒	☐ MRI-based neuroimaging

Antibodies

Antibodies used	HRP-conjugated polyclonal mouse anti-rabbit IgG (Southern Biotech, Cat. No. 4090-05), HRP-conjugated polyclonal goat anti-human IgG (Southern Biotech, Cat. No. 2040-05), CytoGam (CSL Behring, NDC code: 49591-532-51), 2M7 (in house), SM-5 (in house), SM-10 (in house)
Validation	Cytogam (cytomegalovirus immune globulin intravenous, human) are polyclonal antibodies used in clinic and come from controlled sources. Cytogam is a hyperimmune product that contains antibodies specific for the cytomegalovirus.

Eukaryotic cell lines

Policy information about cell lines and Sex and Gender in Research

Cell line source(s)	Expi293F™ (ThermoFisher), HEK293T (ATCC), ARPE-19 (ATCC), HFF-1 (ATCC), THP-1 (ATCC)
Authentication	None
Mycoplasma contamination	Cell lines were negative for mycoplasma contamination
Commonly misidentified lines (See ICLAC register)	*Name any commonly misidentified cell lines used in the study and provide a rationale for their use.*

Animals and other research organisms

Policy information about studies involving animals; ARRIVE guidelines recommended for reporting animal research, and Sex and Gender in Research

Laboratory animals	New Zealand White Rabbit
Wild animals	This study did not involve wild animals.
Reporting on sex	All 18 New Zealand White Rabbits are females.
Field-collected samples	This study did not involve samples collected from the field.
Ethics oversight	The in vivo immunization and sample collection experiments in rabbits were approved by IACUC of Duke University Medical Center (Protocol Registry Number A248-17-11). All experimentation adhered to the USDA Animal Welfare regulations, PHS Policy on Humane Care and Use of Laboratory Animals, the NIH/NRC Guide for the Care and Use of Laboratory Animals, Assessment and Accreditation of Laboratory Animal Care International (AAALAC) accreditation guidelines, and Duke University Institutional Animal Care and Use Committee (DUIACUC) care and use policies.

Note that full information on the approval of the study protocol must also be provided in the manuscript.

Plants

Seed stocks	*Report on the source of all seed stocks or other plant material used. If applicable, state the seed stock centre and catalogue number. If plant specimens were collected from the field, describe the collection location, date and sampling procedures.*
Novel plant genotypes	*Describe the methods by which all novel plant genotypes were produced. This includes those generated by transgenic approaches, gene editing, chemical/radiation-based mutagenesis and hybridization. For transgenic lines, describe the transformation method, the number of independent lines analyzed and the generation upon which experiments were performed. For gene-edited lines, describe the editor used, the endogenous sequence targeted for editing, the targeting guide RNA sequence (if applicable) and how the editor was applied.*
Authentication	*Describe any authentication procedures for each seed stock used or novel genotype generated. Describe any experiments used to assess the effect of a mutation and, where applicable, how potential secondary effects (e.g. second site T-DNA insertions, mosiacism, off-target gene editing) were examined.*

Flow Cytometry

Plots

Confirm that:

☒ The axis labels state the marker and fluorochrome used (e.g. CD4-FITC).

☒ The axis scales are clearly visible. Include numbers along axes only for bottom left plot of group (a 'group' is an analysis of identical markers).

☒ All plots are contour plots with outliers or pseudocolor plots.

☒ A numerical value for number of cells or percentage (with statistics) is provided.

Methodology

Sample preparation	Samples collected from New Zealand White Rabbis were detailed in the manuscript.
Instrument	Spectramax plate reader (Thermo Scientific), ImageXpress Pico (Molecular Devices), FACSDiva, ImmunoSpot® S6 Ultimate M2 Analyzer (Cellular Technology Limited), Genepix 4100A (Molecular Devices)
Software	Excel, Flowjo, CellReporterXpress, ImmunoSpot® Software, Genepix 4100A
Cell population abundance	Cell populations are noted in the representative gating strategies included in the supplemental figures
Gating strategy	Gating strategy is included in the supplementary figures

☒ Tick this box to confirm that a figure exemplifying the gating strategy is provided in the Supplementary Information.
